# β-Nicotinamide adenine dinucleotide (β-NAD) acts as a bronchodilator

**DOI:** 10.1371/journal.pone.0334491

**Published:** 2025-10-14

**Authors:** Innokentij Jurastow, Silke Wiegand, Amir Rafiq, Anna Zakrzewicz, Sandra Engel, Adriano Sanna, Daniel von der Beck, Walter Klepetko, Andreas Hecker, Andreas Günther, Moritz Bünemann, Gabriela Krasteva-Christ, Maryam Keshavarz

**Affiliations:** 1 Institute for Anatomy and Cell Biology, German Center for Lung Research, Justus Liebig University, Giessen, Germany; 2 Excellence Cluster Cardio-Pulmonary Institute, Justus Liebig University, Giessen, Germany; 3 Department of Anesthesiology and Critical Care Medicine, Charité Campus Virchow Klinikum, Berlin, Germany; 4 Department of General and Thoracic Surgery, University Hospital of Giessen, Justus-Liebig-University Giessen, Giessen, Germany; 5 Institute for Pharmacology and Clinical Pharmacy, Faculty of Pharmacy, Philipps-University Marburg, Marburg, Germany; 6 Department of Thoracic Surgery, Vienna General Hospital, Vienna, Austria; 7 Center for Interstitial and Rare Lung Diseases, University of Giessen and Marburg Lung Center, member of the German Center of Lung Research; Cardiopulmonary Institute; Institute for Lung Health; European IPF Registry and Biobank; Lung Clinic Agaplesion Evangelisches Krankenhaus Mittelhessen, Giessen, Germany; 8 Institute of Anatomy and Cell Biology, School of Medicine, Saarland University, Homburg, Germany; 9 Center for Gender-Specific Biology and Medicine (CGBM), Saarland University, Homburg, Germany; 10 PharmaScienceHub (PSH), Saarbrücken, Germany; Cinvestav-IPN, MEXICO

## Abstract

**Introduction:**

β-Nicotinamide adenine dinucleotide (β-NAD) is recognized as a sympathetic neurotransmitter that relaxes vascular and intestinal smooth muscle through purinergic receptor pathways. In the lung, β-NAD has been associated with anti-inflammatory effects, but its role in regulating airway smooth muscle tone remains unexplored. This study investigates the impact of β-NAD on airway smooth muscle and elucidates the underlying mechanisms of its action.

**Materials and methods:**

Airway constriction was assessed as a force in organ bath (mouse trachea, human bronchioli) and as a luminal area in mouse precision-cut lung slices. The latter was combined with recording changes in [Ca^2+^] and membrane potential. Intracellular calcium and cyclic AMP concentrations were recorded in isolated airway smooth muscle cells.

**Results:**

β-NAD did not affect baseline tension/area in the trachea, bronchi, and bronchioli. Airways precontracted with muscarine were concentration-dependently relaxed with β-NAD by up to 100%, being as effective as salbutamol. The airway relaxing effect of β-NAD was resistant to purinergic inhibitors, to inhibition of G_s_- and G_i_-signaling, and insensitive to several other blockers of common relaxation pathways. Isolated airway smooth muscle cells and bronchial smooth muscle in precision-cut lung slices responded to β-NAD with increased [Ca^2+^]_i_ and depolarization of the cell membrane while relaxing. β-NAD increased intracellular cAMP levels in airway smooth muscle. *In silico* analysis revealed low expression of soluble adenylyl cyclase (ADCY10) in mouse and human airway smooth muscle, consistent with the lack of effect of the sAC inhibitor KH7 and preserved responses in sAC-deficient mice. These findings implicate transmembrane adenylyl cyclases as the likely cAMP source. Phosphodiesterase-4 inhibition with rolipram enhanced β-NAD-induced relaxation, suggesting a role for compartmentalized cAMP signaling.

**Conclusions:**

Extracellular β-NAD relaxes airway smooth muscle via a noncanonical, cAMP-linked pathway that is independent of classical Gi- and Gs-coupled receptor signaling. This pathway is enhanced by PDE4 inhibition and likely involves localized cAMP pools generated by transmembrane adenylyl cyclases. These findings identify β-NAD as a potential modulator of airway tone and support further exploration of its physiological and therapeutic relevance.

## Introduction

Bronchoconstriction and inflammation are hallmarks of chronic obstructive pulmonary disease (COPD) and asthma [1–5]. These processes are regulated by complex signaling networks that involve airway smooth muscle contraction and immune cell activation [6]. While bronchoconstriction is mediated through pathways involving muscarinic acetylcholine receptors, bronchodilation is regulated by β₂-adrenoreceptors and inflammation is driven by immune cell recruitment and cytokine release [6,7].

Airway smooth muscle (ASM) is a critical target for bronchodilators, as its contraction in response to various stimuli drives bronchoconstriction, which significantly contributes to the airflow limitation and airway hyperresponsiveness characteristic of COPD and asthma [4,5]. Given the dynamic interplay between inflammation and ASM contraction, molecules that influence both processes may have important implications for airway regulation.

β-Nicotinamide adenine dinucleotide (β-NAD), a ubiquitous coenzyme synthesized from niacin or tryptophan, plays a crucial role in cellular metabolism and redox reactions [8]. It is also recognized as a vital regulator of immune homeostasis and inflammation, influencing various physiological and pathological processes [9,10]. β-NAD has been shown to exert anti-inflammatory effects in the lung, specifically by blocking neutrophil infiltration, reducing edema formation, and dampening the upregulation of proinflammatory cytokines following lipopolysaccharide challenge or lung transplantation [11,12]. Exogenous β-NAD has been shown to modulate both innate and adaptive immune responses, indicating its therapeutic potential in diverse inflammatory and oxidative stress-related conditions [10,13–15].

Beyond its metabolic and anti-inflammatory properties, β-NAD can be released from autonomic nerve fibers to relax smooth muscle in various tissues, including the vasculature, gastrointestinal tract, and urinary bladder, through purinergic P2Y1 and P2Y11 receptor-mediated signaling [16–19].

This study investigates whether extracellular β-NAD exerts bronchodilatory effects in murine and human ASM using preclinical models, including organ bath experiments and precision-cut lung slices (PCLS). To explore the underlying mechanisms, we recorded changes in intracellular cyclic adenosine monophosphate (cAMP) and calcium concentration ([Ca²⁺]ᵢ) in isolated bronchial smooth muscle cells, as well as simultaneous alterations in [Ca²⁺]ᵢ, membrane potential, and bronchial luminal area in PCLS. Our findings suggest that β-NAD induces potent bronchodilation by relaxing precontracted airways through mechanisms that are independent of classical Gi- and Gs-coupled receptor signaling, accompanied by membrane depolarization and an increase in intracellular calcium and cAMP levels. Notably, this relaxation appears to involve mechanisms distinct from traditional purinergic and adenosine receptor pathways. Additionally, phosphodiesterase inhibition with rolipram enhanced β-NAD-induced relaxation. Given its combined anti-inflammatory and antioxidative properties, β-NAD emerges as a promising candidate for novel therapeutic approaches in asthma and COPD, though further studies are needed to fully elucidate its precise mechanisms of action.

## Materials and methods

### Mice

Specific-pathogen-free C57Bl/6N mice (Jackson Laboratories, aged 8–20 weeks), mice with genetic deletion of the C1 domain of soluble adenylyl cyclase (SAC) (B6;129S5-*Sacy*^*tm1Lex*^) [20] and corresponding wildtype (aged 43–48 weeks), and mice with genetic deletion of the SAC C2 domain (B6;129Sv-Adyc10^tm1.1Geno^/Geno) [21] and wildtype littermates (aged 17–25 weeks) of both sexes were used. Animals were anesthetized by isoflurane (5%) (Abbott) and euthanized by cervical dislocation or transecting the inferior vena cava. All animals were handled in compliance with the guidelines set by the European Community for the care and use of animals, as well as the U.S. Animal Welfare Act. The breeding and use of samples from euthanized mice for in vitro experiments were approved and registered with the relevant authorities at the Regierungspräsidium Giessen (Hesse, Germany) under registration numbers #A5/2010, #A61/2012, JLU 579_M, and #1244.

### Human samples

Human lung tissue was obtained from four patients with COPD and idiopathic pulmonary fibrosis (IPF) undergoing lung transplantation at the University Hospitals of Vienna Medical University and Justus Liebig University. Between 14.06.2013 and 25.02.2014, patients were enrolled in the study. Tissue collection was conducted with written informed consent from each patient as part of the European IPF Register (eurIPFreg). The data safety protocol and informed consent process of eurIPFreg have been reviewed and approved by multiple European ethical committees, including the JLU Giessen Ethics Committee (AZ 111/08). Lung tissue explantation generally took place 12–24 hours before the experiments.

### Chemicals and reagents

All chemicals and reagents used in this study are listed in S1 Table.

### Videomorphometry of murine PCLS with fluorimetric recording of [Ca^2+^]_i_ and membrane potential

Videomorphometric recordings from PCLS were performed as described previously [22–24]. Mice were killed by cervical dislocation. Airways were filled via a tracheal cannula with 1.5% low-melting point agarose in HEPES-Ringer buffer consisting of 120 mM NaCl, 5 mM KCl, 2 mM CaCl_2_, 1 mM MgCl_2_, 25 mM NaHCO_3_, 5.5 mM HEPES, and 1 mM d-glucose, filtered through 0.22 micron filter. Lungs and heart were removed *en bloc* and transferred into ice-cold HEPES-Ringer buffer. Lung lobes were sectioned into 200 μm thick slices using a vibratome (VT1000S, Leica, Wetzlar, Germany) and incubated for at least 2 h at 37°C in phenolred-free minimal essential medium (MEM, Thermo Fisher Scientific, Waltham, MA USA) continuously bubbled with 21% O_2_, 5% CO_2_, 74% N_2_. For videomicroscopic recordings, PCLS were transferred to a flow-through superfusion chamber (Hugo Sachs Elektronik, March-Hugstetten, Germany) mounted on an inverted microscope (DM IL, Leica) and fixed using a nylon mesh. Basal luminal area was assessed after 5 min perfusion with MEM at a flow rate of 0.7 ml/min. Drugs were applied at flow arrest. Images of bronchi were recorded every 1 min using a charge-coupled device-camera (Stemmer Imaging, Puchheim, Germany). Changes in the bronchial luminal area were evaluated by Optimas software (Optimas, Version 6.5, Stemmer, Puchheim, Germany). Basal bronchial luminal area was set as 100%, and constriction or dilatation was expressed as a relative decrease or increase in this area, respectively. Only those bronchi responding to 60 mM KCl or muscarine (10 µM) with at least 20% reduction of their luminal area were included in the final data analysis.

For the simultaneous recording of [Ca^2+^]_i_, membrane potential and bronchial luminal area, PCLS were incubated for 40 min in MEM containing the Ca^2+^ indicator Calcium Orange AM (5 µM), the membrane potential sensitive dye DiBAC(4)3 (5 µM) and the organic anion transport inhibitor sulfobromophthaleine (100 µM). [Ca^2+^]_i_ was monitored with a confocal laser scanning microscope (LSM 710, Zeiss; imaging speed 1.57 frames/s; excitation at 549 nm wavelength (*λ*), recording at *λ* = 576 nm for Calcium Orange, *λ* = 494 nm and *λ* = 517 nm, respectively, for DiBAC4(3))*.* Test stimuli and concentrations were 10 µM muscarine and 1 mM β-NAD. Regions of interest were selected in individual smooth muscle cells and adjusted in each frame manually to track the Ca^2+^-signal in the same cells when they were contracting.

### Force recordings of explanted murine trachea and human bronchioli

Murine tracheal segments were recorded as described earlier [24–26]. Mice were killed by exsanguination and the middle segment of the trachea, comprising four cartilage rings, was explanted. Isometric contraction was measured in isolated rings that were mounted between two stainless steel clips in vertical 15 ml organ baths of a computerized isolated organ bath system (ADInstruments GmbH, Heidelberg, Germany).

The chamber was filled with 37°C warm MEM, which was supplemented with 1% penicillin/streptomycin and continuously aerated with a 95% O_2_/5% CO_2_ gas mixture. The temperature was held at 37°C using a bath circulator (Thermo Fisher Scientific, Waltham, USA). The upper stainless clip was connected to an isometric force transducer (Power Lab 8.30; ADInstruments GmbH, Heidelberg, Germany). Tissues were equilibrated against a passive load of 1 g for all rings. After this period, samples were adjusted at 0.5 g tension. Changes in the isometric contraction were converted by the transducer into an amplified direct current output voltage and assigned to the software LabChart 6 (ADInstruments GmbH, Heidelberg, Germany). All samples were equilibrated for 30 min until they reached a stable baseline tension.

When the relaxing effect of β-NAD was tested, segments were pre-contracted with muscarine (10 µM), KCl (60 mM), or by electrical field stimulation (EFS; 10 Hz, 10 V, 2 ms for 6 min; 3165 Multiplexing Pulse Booster; Ugo Basile, Gemonio, Italy) before adding β-NAD (0.1–7 mM). The drugs used, along with their respective targets and functions, are listed in S2 Table.

Different vehicles were used for the various substances, and the experimental setups varied to evaluate the β-NAD-induced relaxation effect. The relaxation induced by β-NAD was calculated as a percentage of the muscarine-induced contraction. In the first setup, 1 mM β-NAD was applied, with blockers introduced 15 min before the addition of muscarine. The substances tested included vehicle (0.02% DMSO), 8-sPT (8-(p-sulfophenyl) theophylline, 10 µM), apamin (10 µM), Rp-cAMP (Rp-cyclic adenosine 3’,5’-phosphorothioate, 100 µM), and a purinergic receptor blocker cocktail (P-Block) consisting of suramin (100 µM), pyridoxalphosphate-6-azophenyl-2’,4’-disulfonic acid (PPADS, 10 µM), and MRS2179 (10 µM).

In another setup, 5 mM β-NAD was applied after pretreatment with blockers that were administered 50 min before muscarine. Substances tested included vehicle (PBS), cholera toxin (CTX, 2 ng/ml), pertussis toxin (PTX, 5 ng/ml), and L-NAME (L-NG-nitroarginine methyl ester, 100 µM).

In a third setup, 5 mM β-NAD was applied, with blockers introduced 5 min after muscarine and 10 min before β-NAD. Substances tested in this protocol included vehicle (0.02% DMSO), rolipram (100 µM), and FPL64176 (10 µM).

Muscarine-induced contractions, including those seen after using blockers, were compared to their normal control responses with vehicles. Data from the first setup were primarily used to evaluate muscarine-induced contractions, except for rolipram (100 µM) and FPL64176 (10 µM), which were applied after the muscarine response in their respective setups. Additional experiments were performed to determine the effects of these substances on contraction.

In the case of U73122, the muscarine response was completely inhibited, preventing the measurement of β-NAD-induced relaxation. Here, U73122 (10 µM) was applied 15 minutes before muscarine, with vehicle (0.02% DMSO) serving as a control. These designed protocols ensured the assessment of relaxation and contraction effects across varying experimental conditions.

Human bronchioli with a diameter of 1.5–2 mm were dissected at a length of 5 mm and treated and evaluated as described above for murine trachea.

### Isolation and culture of murine tracheal smooth muscle cells

Explanted tracheae (n = 4) were enzymatically digested in papain (2 mg/ml), bovine serum albumin (BSA; 2 mg/ml), dithiothreitol (DTT; 0.5 mg/ml) and L-cysteine (25 mM) in physiological salt solution (PSS; containing in [mM]: NaCl 140, KCl 5, MgCl₂ 1, HEPES 10, glucose 10 and 1 mM sodium pyruvate; pH adjusted to 7.4)for 30 min at 37°C, centrifuged (200 × g, 5 min) and mechanically dissociated. Inactivation of papain by leupeptin (2 µl/ml) was followed by a second enzymatic digestion in Dispase II (2 mg/ml), BSA (2 mg/ml), DTT (1 mg/ml) in PSS for 1 h, centrifugation, and mechanical dissociation. The tissue was then centrifuged (200 × g, 5 min) and resuspended in PSS. Cells were cultured in medium (300 µl RPMI + 10% fetal calf serum + 1% penicillin/streptomycin) and grown for experiments on coverslips coated with fibronectin (1 mg/ml) (~20,000 cells per coverslip).

### Culture of human bronchial smooth muscle cells (HBSMC)

HBSMC (C-12561, PromoCell, Heidelberg, Germany) were cultured in Smooth Muscle Cell Growth Medium 2 for at least 24 h at 37°C. One day before experiments, cells were detached with 100 μl trypsin/EDTA solution (0.5 g/l trypsin and 0.2 g/l EDTA•4Na) per cm^2^ of vessel surface at room temperature. Cells were centrifuged (200 × g, 5 min) and seeded on coverslips coated with fibronectin (1 mg/ml).

### Measurement of [Ca²⁺]i in isolated murine tracheal smooth muscle cells, HBSMC, and in the M3WT4 cell line

Freshly isolated murine tracheal smooth muscle cells or cultured HBSMC were loaded with Fura-2 AM (10 µM) and the organic anion transport inhibitor sulfobromophthalein (100 µM) for 20 min at 37°C. Coverslips with cells were transferred into a delta-T-dish (Bioptechs, Butler, PA, USA) on a stage of an inverted light microscope with a temperature-controlled stage (Olympus BX50WI, Olympus, Hamburg, Germany) and constantly perfused with warmed PSS. The measurements were performed at a constant temperature of 37°C. Test stimuli and concentrations were 1 mM for β-NAD and 100 µM for ATP, which served as a positive control at the end of the experiment. Fura-2 was excited at 340 and 380 nm wavelengths with a monochromator (Polychrome II, TiLL Photonics, Gräfelfing, Germany), and fluorescence intensity was recorded at λ > 420 nm with a slow-scan charge-coupled device camera system (Kamera IMAGO, C11440 Orca-flash 4.0 Hamamatsu, TiLL Photonics). Each cell was tracked independently and the fluorescence intensity ratio at 340/380 nm excitation was analyzed with image analysis software (TiLL Vision, TiLL Photonics). At the end of the experiments, cells were immunolabeled for α-smooth muscle actin for 1 h with the FITC-conjugated mouse monoclonal antibody 1A4 (2 µg/ml; Sigma Aldrich), and evaluated by epifluorescence microscopy (Axioplan 2 imaging, Zeiss, Jena, Germany) to validate that measurements were done on smooth muscle cells.

M3WT4 cells (CHO-K1 cells expressing rat M3 muscarinic acetylcholine receptor, ATCC Cat#CRL-1981) were freshly thawed for each experiment and seeded directly in Ham’s F-12K (Kaighn’s) medium supplemented with 10% FBS, 1% penicillin/streptomycin on laminin and poly-L-lysine coated glass coverslips (20,000 cells per coverslip). The attachment of cells was achieved by incubating for 30–90 min. Cells were then loaded for 60–90 min with Fura 2-AM (4 µM) in Ham’s F-12K (Kaighn’s) medium. Changes in intracellular calcium concentration [Ca^2+^]_i_ were recorded with a slow-scan charge-coupled device camera system (Kamera IMAGO, C11440 Orca-flash 4.0 Hamamatsu, TiLL Photonics) for the ratiometric recording of single cells. Coverslips were transferred into a Bioptechs Delta T dish and temperature (37ºC) was controlled during the experiment. Cells were constantly perfused with Locke buffer (in mM: 136 NaCl, 5.6 KCl, 1.2 MgCl_2_, 2.2 CaCl_2_, 1.2 NaH_2_PO_4_, 14.3 NaHCO_3_ and 10 mM dextrose, 37ºC, pH 7.4). Changes in intracellular calcium concentration ([Ca²⁺]ᵢ) were recorded every second as described above. After application of β-NAD (1 mM), coverslips were washed for 3 min with Locke buffer, followed by addition of acetylcholine (10 µM) as a positive control. [Ca²⁺]ᵢ responses were quantified as changes relative to the average baseline fluorescence measured during the 15 seconds before acetylcholine application. Only cells that responded to acetylcholine were included in the analysis.

### Transfection of HBSMC and FRET measurement of cAMP concentration

Primary HBSMC were transfected with a plasmid encoding a fluorescence resonance energy transfer (FRET)-based cAMP indicator, i.e., human exchange protein directly activated by cAMP (EPAC1) (GenBank™ accession number AF103905) flanked by the green fluorescent protein variants enhanced yellow fluorescent protein (EYFP) and enhanced cyan fluorescent protein (ECFP) respectively (E1). Transfection was done by electroporation, using the Basic Nucleofector™ Kit for primary mammalian smooth muscle cells (SMC) (Lonza, Cologne, Germany) and the Nucleofector device II (Lonza) according to the manufacturer’s instructions. Cells were transfected with 2 µg of the construct per 1x10^6^ cells using program U-025.

FRET measurements of transiently transfected HBSMC were performed approximately 48–54 h after transfection at room temperature (20–24°C) using an inverted microscope (Eclipse Ti, Nikon, Duesseldorf, Germany) equipped with a 100x oil immersion objective (Plan Apo VC 100x/1.40 oil ∞ /0.17 Dic N2, Nikon, Duesseldorf, Germany). A fast-switching xenon arc-based illumination system (Lambda DG-4, Sutter Instrument, Hofheim, Germany) was used as a light source. The following filters (all from Chroma, Olching, Germany) were used: ET 430/24× (for ECFP excitation) or ET 500/20× (for EYFP excitation);, T455LP (long-pass beamsplitter to collect combined fluorescence of CFP and YFP); 59017bs and 59017m (CFP/YFP beamsplitter and emission filter); z488/800–1064rpc (beamsplitter); ET 480/40 (CFP emission) and HC 534/20 (YFP emission). The last three components were set in an Optosplit II (Cairn Research, Faversham, UK) to simultaneously record CFP and YFP fluorescence using a fast CCD camera (Evolve512, Roper Scientific, Martinsried, Germany). Microscope, camera and DG-4 were controlled by NIS-Elements AR software (Laboratory Imaging, Praha, CZ). To synchronize the camera and lamp, an additional triggerbox was supplied by Nikon. Cells were continuously superfused with HEPES-buffered saline (137 mM NaCl, 5.4 mM KCl, 2 mM CaCl_2_, 1 mM MgCl_2_ and 10 mM HEPES, pH 7.3) or buffer containing agonists using a fast-switching eight-channel solenoid valve-controlled pressurized perfusion system (Ala-VC3–8SP, ALA Scientific Instruments, Framingdale, US). For FRET measurements, CFP and YFP emissions were recorded simultaneously while cells were excited with 430 nm light. Depending on the fluorescence intensity, the illumination time was set to 20–40 ms at an interval of 500 ms or 2 s. The lamp was set to the lowest intensity to prevent bleaching. Cell fluorescence was recorded at 488 ± 20 nm (F488 for CFP) and 534 ± 10 nm (F534 for YFP) and corrected for background fluorescence, resulting in FCFP and FYFP values. To determine FRET, FYFP was additionally corrected for bleed-through of CFP fluorescence into the F534 channel and direct excitation of YFP at 430 ± 12 nm excitation was subtracted. The resulting fluorescence was divided by FCFP, and the FRET ratio (FYFP/FCFP) was calculated.. Agonists used in the experiments were β-NAD (1 mM) and isoproterenol (10 µM).

### ScRNA data set analysis

Three single cell sequencing datasets were downloaded from the National Centre for Biotechnology Information Gene Expression Omnibus (NCBI GEO): two from human airways [27] (GSE136831 and GSE134174) and one from mouse airways [28] (GSE244215). From the human datasets, all cells/nuclei preparations belonging to the tracheal tissue were extracted and subdivided into epithelial (epithelial cell adhesion molecule, EPCAM > 1.0) and smooth muscle cells (smooth muscle actin, ACTA2 > 1.0). For the mouse dataset, both young (2 months old) and old mice (24 months old) were analysed to evaluate whether age-related changes in gene expression could occur. Cells were then subdivided into epithelial (Epcam > 1.0) and smooth muscle cells (Acta2 > 1.0).

The mRNA level of the following genes under the different conditions was evaluated: *Epcam*, *Acta2*, adenylate cyclase isoforms (*Adcy1–Adcy10* in mouse, *ADCY1–ADCY10* in human), protein kinase cAMP-activated catalytic subunit alpha (*Prkaca*/ *PRKACA*) and beta (*Prkacb*/ *PRKACB*), protein kinase cAMP-dependent type I regulatory subunit alpha (*Prkar1a*/ *PRKAR1A*) and beta (*Prkar1b*/ *PRKAR1B*), protein kinase cAMP-dependent type II regulatory subunit alpha (*Prkar2a*/ *PRKAR2A*) and beta (*Prkar2b*/ *PRKAR2B*), Rap guanine nucleotide exchange factor 3 (*Rapgef3*/ *RAPGEF3*) and 4 (*Rapgef4*/ *RAPGEF4*), phosphodiesterase 4A (*Pde4a*/ *PDE4A*), 4B (*Pde4b*/ *PDE4B*), and 4D (*Pde4d*/ *PDE4D*), ADP-ribosyl cyclase 1 (*Cd38*/ *CD38*), bone marrow stromal cell antigen 1 (*Bst1*/ *BST1*), sirtuin isoforms 1–7 (*Sirt1–Sirt7*/ *SIRT1–SIRT7*), ryanodine receptor isoforms 1–3 (*Ryr1–Ryr3*/ *RYR1–RYR3*), inositol 1,4,5-trisphosphate receptor types 1–3 (*Itpr1–Itpr3*/ *ITPR1–ITPR3*), and two-pore segment channel 1 (*Tpcn1*/ *TPCN1*) and 2 (*Tpcn2*/ *TPCN2*).

For in silico analysis, the following libraries were used: anndata, Jupyter-Lab, pandas and scanpy [29]. Generation of the co-expression matrices was performed for each airway subset (human epithelial cells/nuclei preparations, human smooth muscle cells/nuclei preparations, mouse epithelial cells, and mouse smooth muscle cells), considering the number of cells expressing each of the above-mentioned genes with a value > 1.0.

### Statistical analysis

All experiments were performed using randomly assigned mice. All data points and “n” values reflect biological replicates (i.e., mice or cells, or tissues). For the videomorphometry analysis, the data are presented as n = number of PCLS/number of mice. For human organ bath experiments, *n* refers to the number of bronchioli, followed by the number of donor lungs from which they were obtained (bronchioli/lungs). Reproducibility was verified by replicating experiments as specified in the respective figure legends. All experimental findings described here were reliably reproduced as seen in the scatterplots depicting all individual data points and means ± SEM. Data in the graphs depicting time courses or concentration responses are presented as means ± SEM. Statistical analysis was conducted as described in the figure captions using Prism 9 (GraphPad Software). Data normality was assessed with the Kolmogorov-Smirnov test, which indicated a deviation from a normal distribution. Consequently, all data were analyzed using nonparametric statistical tests: the Mann-Whitney U test for two-group comparisons and the Kruskal-Wallis test followed by Dunn’s multiple comparisons test for multiple groups. For the analysis of intracellular cAMP concentration in HBSMC via FRET using a paired Wilcoxon signed-rank test (two-tailed) with Bonferroni correction for multiple comparisons. No statistical methods were used to predetermine the sample size. Differences were considered statistically significant when P ≤ 0.05.

## Results

### β-NAD relaxes constricted murine airways

**Fig 1:**
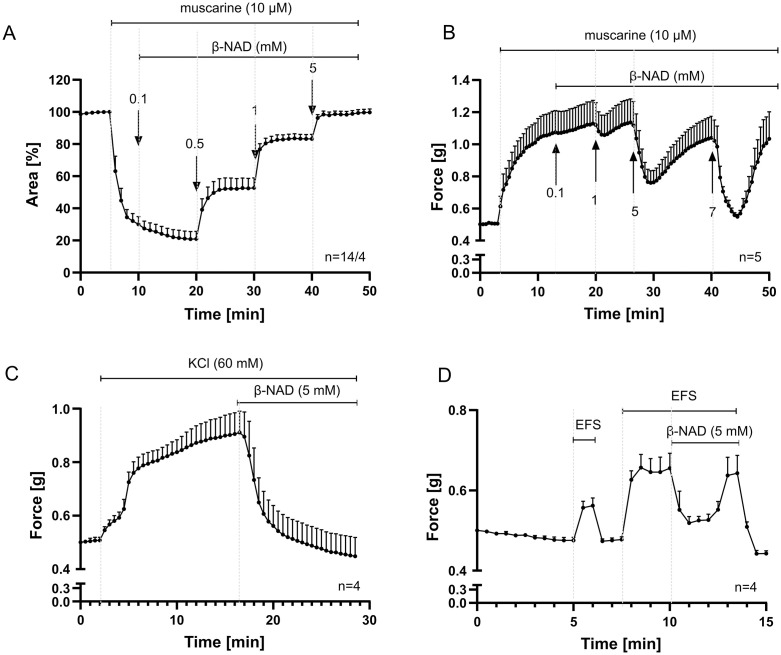
β-NAD relaxes murine airways precontracted with muscarine. Videomorphometric recording of luminal bronchial area in PCLS (A) and force recording from trachea in organ bath (B- D). β-NAD concentration-dependently counteracts constriction evoked by muscarine. In the trachea, the relaxing effect does not persist over time (B). Force recordings from tracheal preparations in an organ bath, induced by KCl depolarization (C) and electrical field stimulation (EFS; 10 Hz, 10 V, 2 ms) (D). EFS is commonly used to activate nerve-mediated contractions, although no pharmacological confirmation was performed. The initial EFS was applied to verify tissue viability. n indicates the number of animals, or in the case of PCLS, the number of airways studied, followed by the number of animals used for PCLS preparation, presented as airway/mice. Data are expressed as mean ± SEM.

**Fig 2:**
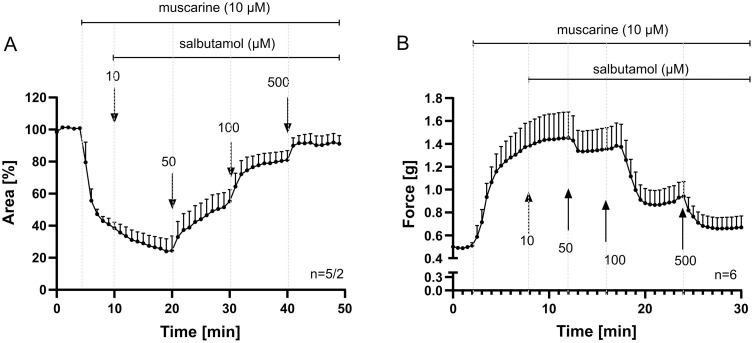
Salbutamol relaxes murine airways precontracted with muscarine. The relaxing effect of the β-adrenoreceptor agonist salbutamol is shown for comparison. Videomorphometric recording of luminal bronchial area in PCLS (A) and force recording from trachea in organ bath (B). Salbutamol concentration-dependently counteracts constriction evoked by muscarine. n indicates the number of animals, or in the case of PCLS, the number of airways studied, followed by the number of animals used for PCLS preparation, presented as airway/mice. Data are expressed as mean ± SEM.

β-NAD did neither alter the baseline diameter of bronchi in PCLS nor the tension of the explanted trachea (S1A and S1B Fig). However, it effectively relaxed murine bronchi and trachea precontracted with 10 µM muscarine (Fig 1A and 1B), as well as trachea precontracted with 60 mM KCl or electrical field stimulation (EFS) (Fig 1C and 1D). EFS is widely used to elicit nerve-mediated contractions in airway tissues, and while we did not include pharmacological controls to confirm this, our observations are consistent with transmitter release from nerve fibers as described in previous studies [30]. At concentrations that produced similar levels of bronchial relaxation (1 mM β-NAD and 100 µM salbutamol, selected based on results from Figs 1 and 2), β-NAD was as effective as the β₂-adrenoreceptor agonist salbutamol and achieved maximal relaxation in a faster time course (S2 Fig). Bronchi, assessed by video morphometric analysis of PCLS, appeared more responsive to β-NAD than tracheal preparations studied in organ bath experiments. In tracheae pre-contracted with muscarine, 5 mM β-NAD was required to reduce contractile force by more than 50%, whereas in bronchi, 1 mM β-NAD almost completely reversed bronchoconstriction (>90%). This bronchodilation persisted throughout the observation period, while in the tracheae, contractile tone gradually returned within 5 minutes despite continued exposure to β-NAD. In tracheae pre-contracted with KCl, β-NAD-induced relaxation was maintained over the 10-minute recording period, although longer-term effects were not assessed (Fig 1).

### β-NAD relaxes murine airway smooth muscle independent of G protein-coupled receptors and canonical pathways

**Fig 3:**
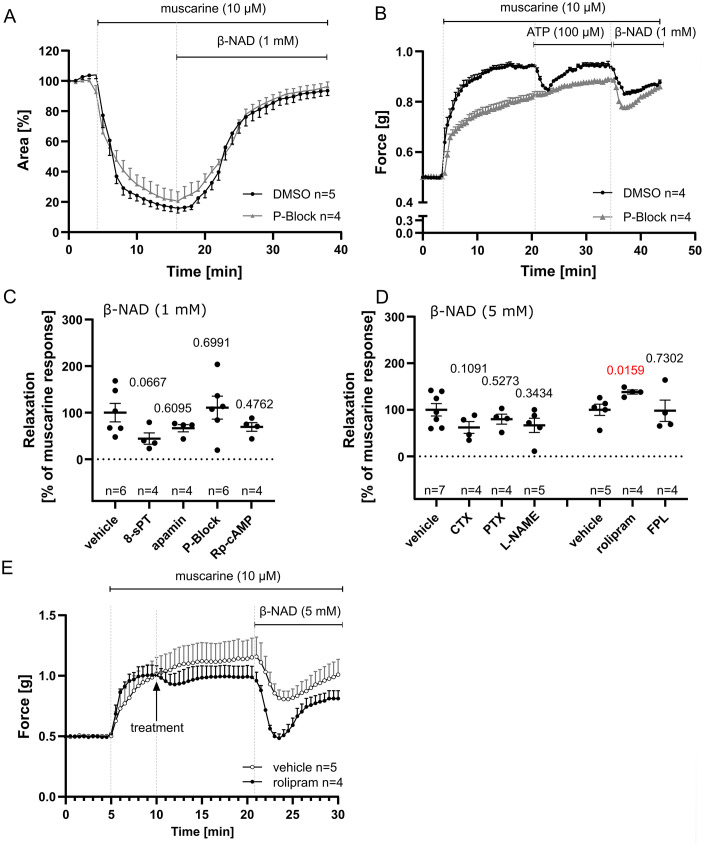
β-NAD relaxes murine airway smooth muscle independent of G-protein coupled receptors and canonical relaxation pathway. (A) Videomorphometric recording of the luminal murine bronchial area in PCLS. (B) Force recording from the trachea in an organ bath setup. Gray lines represent data from preparations pretreated with a purinergic receptor inhibitor cocktail (P-Block), consisting of suramin (100 µM), pyridoxalphosphate-6-azophenyl-2’,4’-disulfonic acid (PPADS, 30 µM), and MRS2179 (10 µM). The P-Block cocktail abrogated ATP-induced tracheal dilatation (B) but did not affect β-NAD-induced relaxation (A). DMSO indicates dimethyl sulfoxide (final concentration 0.03%), used as a vehicle control. n denotes the number of animals used. (C) Effect of inhibitors on β-NAD-induced relaxation of muscarine-precontracted tracheal segments. Relaxation induced by β-NAD is expressed as a percentage of the muscarine-induced contraction. Data are normalized to vehicle control for each experimental setup. Numbers above data points indicate p-values (compared to the respective vehicle control), significant p-values (< 0.05) shown in red; Kruskal-Wallis test followed by Dunn’s multiple comparisons test. Error bars represent the mean ± SEM. Substances tested include vehicle controls (C: 0.03% DMSO, PBS; D: 0.01% DMSO, 0.02% DMSO), purinergic receptor inhibitors (8-sPT, 10 µM; and P-Block comprising suramin, 100 µM, and PPADS, 30 µM), the small-conductance calcium-activated potassium (SK) channel blocker apamin (10 µM), the protein kinase A (PKA) inhibitor Rp-cAMP (100 µM), cholera toxin (CTX, 2 ng/ml), pertussis toxin (PTX, 5 ng/ml), the nitric oxide synthase inhibitor L-NAME (100 µM), the phosphodiesterase-4 (PDE4) inhibitor rolipram (100 µM), and the L-type Ca² ⁺ channel activator FPL 64176 (10 µM). Rolipram significantly enhanced relaxation (control: 99.99 ± 11.92; Rolipram: 138.13 ± 4.41; p = 0.0159). (E) Representative traces showing the effects of rolipram pretreatment on β-NAD-induced tracheal relaxation in murine airways.

In vascular, intestinal, and urinary bladder smooth muscle, β-NAD is assumed to exert its relaxant effect through purinergic P2Y1 and P2Y11 receptor signaling [16–19]. However, a purinergic receptor inhibitor cocktail consisting of suramin (100 µM), PPADS (30 µM), and MRS2179 (10 µM) did not affect β-NAD-induced tracheal and bronchial relaxation, despite blocking ATP-induced dilatation (Figs 3A–3C and S3).

Blocking Gi and Gs protein signaling using PTX (5 ng/ml) and CTX (2 ng/ml), respectively, also failed to influence β-NAD-induced relaxation (Fig 3D). In contrast, CTX significantly reduced salbutamol-induced relaxation of precontracted tracheal muscle (control: 99.67 ± 0.18; CTX: 62.88 ± 8.57; *p* = 0.0095) (S4 Fig). β-NAD-induced relaxation was insensitive to NO-synthase blockade by L-NAME (100 µM), activation of non-dihydropyridine calcium channels by FPL 64176 (10 µM, used to enhance calcium influx), inhibition of adenosine receptors by 8-(p-sulfophenyl)theophylline (10 µM), inhibition of protein kinase A (PKA) by Rp-cAMP (100 µM) and inhibition of potassium channels by apamin (10 µM) (Fig 3C and 3D). In contrast, it was enhanced by the phosphodiesterase-4 inhibitor rolipram (100 µM) (Fig 3D and 3E). Among all these compounds, 8-sPT, apamin, and Rp-cAMP augmented the contraction induced by muscarine, whereas all others had no significant effect (S5 Fig).

### β-NAD induces a rise in [Ca^2+^]_i_ and depolarization in murine ASM

**Fig 4:**
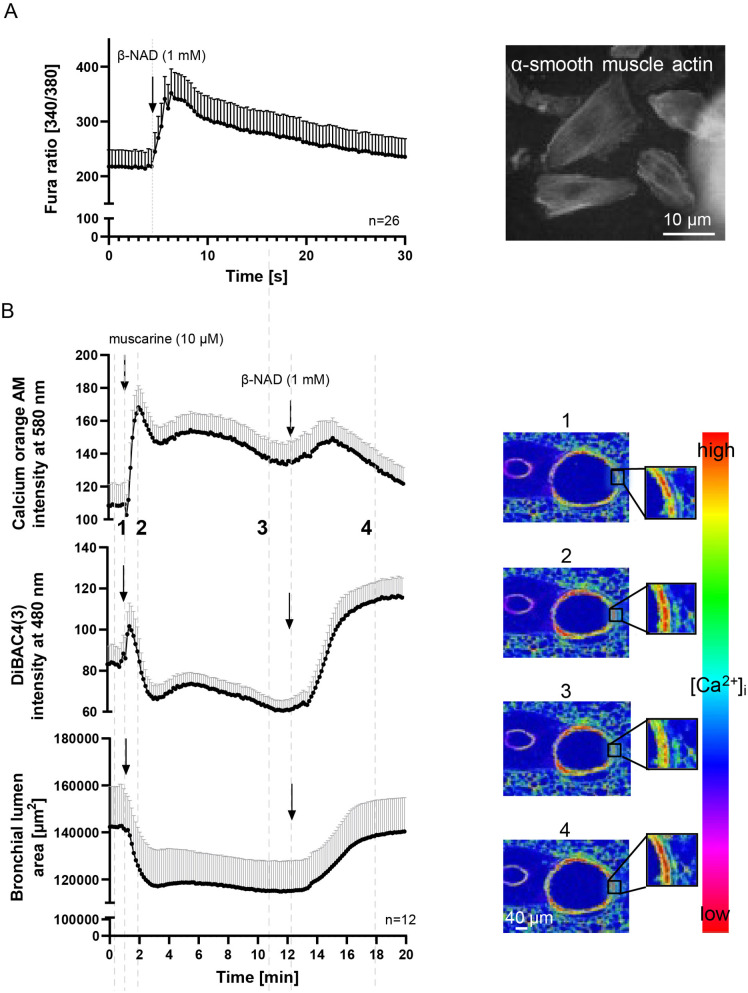
β-NAD causes [Ca^2+^]_i_ increase and depolarization along with bronchorelaxation. (A) Isolated murine tracheal smooth muscle cells were loaded with Fura-2 AM to record changes in intracellular calcium concentration ([Ca²⁺]ᵢ) following the application of 1 mM β-NAD. Cells were subsequently immunolabeled for α-smooth muscle actin to confirm smooth muscle identity (filamentous resolution not visualized under the applied imaging settings). N = 26 cells from 3 independent experiments.(B) Simultaneous recordings of [Ca²⁺]ᵢ (Calcium Orange fluorescence intensity), membrane potential (DiBAC4(3) fluorescence intensity), and bronchial luminal area in PCLS. False-colored PCLS images (right panel) show [Ca²⁺]ᵢ at time points 1–4 as indicated in the left panel curve. Muscarine-induced bronchoconstriction was accompanied by an increase in [Ca²⁺]ᵢ and transient depolarization, while β-NAD-induced bronchodilation was associated with a transient rise in [Ca²⁺]ᵢ, followed by a decline, and a delayed but sustained depolarization. N = 12 denotes the number of ROIs analyzed, taken from 7 PCLS from 4 animals.

An increase in [Ca²⁺]_i_ was observed in cultured murine tracheal smooth muscle cells in response to β-NAD (1 mM) (Fig 4A). Addition of NAD to a cell line stably expressing the M3 muscarinic acetylcholine receptor (M3WT4 cells) did not cause a rise in the Fura-2 340/380 ratio, whereas the positive control acetylcholine did (S6 Fig), demonstrating that the recorded signal did not originate from β-NAD or its reduced form itself.

β-NAD also induced [Ca^2+^]_i_ rise in ASM of murine intrapulmonary bronchi in PCLS analyzed by CLSM and utilizing the Ca^2+^ indicator Calcium Orange AM. This increase occurred simultaneously with ASM depolarization recorded by the voltage-sensitive fluorescent dye DiBAC4(3) and bronchodilation (Fig 4B). Bronchoconstriction evoked by muscarine was accompanied by a rapid increase in [Ca^2+^]_i_ followed by a plateau phase, whereas depolarization was only transient (Fig 4B).

### β-NAD relaxes small human airways and increases [Ca^2+^]_i_ in primary human bronchial smooth muscle cells

**Fig 5:**
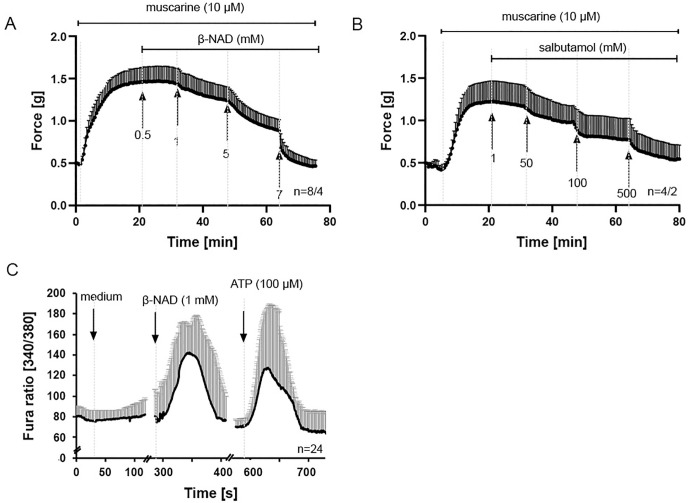
β-NAD induces relaxation of human bronchioli and causes rise in [Ca^2+^]_i_ in HBSMC. (A, B) Force recordings in organ bath, β-NAD (A) and salbutamol (B) concentration-dependently relax precontracted human bronchioli. n refers to the number of bronchioli, followed by the number of lungs from which they were taken, presented as bronchioli/lungs. (C) Recording of [Ca^2+^]_i_ in HBSMC with Fura-2 AM. Both β-NAD and ATP cause rise in [Ca^2+^]_i_. n refers to the number of cells, taken from 4 independent experiments.

β-NAD concentration-dependently relaxed preconstricted (10 µM muscarine) human bronchioli (Fig 5A). In contrast to the murine trachea, where tone returned after initial relaxation (cf. Fig 1B), dilation in human bronchi remained stable throughout the observation period. At the highest concentration tested (7 mM), β-NAD induced a relaxing effect that was at least as pronounced as that of salbutamol (500 µM) (Fig 5B). Consistent with findings in isolated and intact murine ASM, β-NAD induced an increase in [Ca²⁺]_i_ in cultured human bronchial smooth muscle cells, with ATP serving as a positive control (Fig 5C).

### β-NAD increases intracellular cAMP concentration via soluble adenylyl cyclase, but this pathway is not essential for the relaxing effect

**Fig 6:**
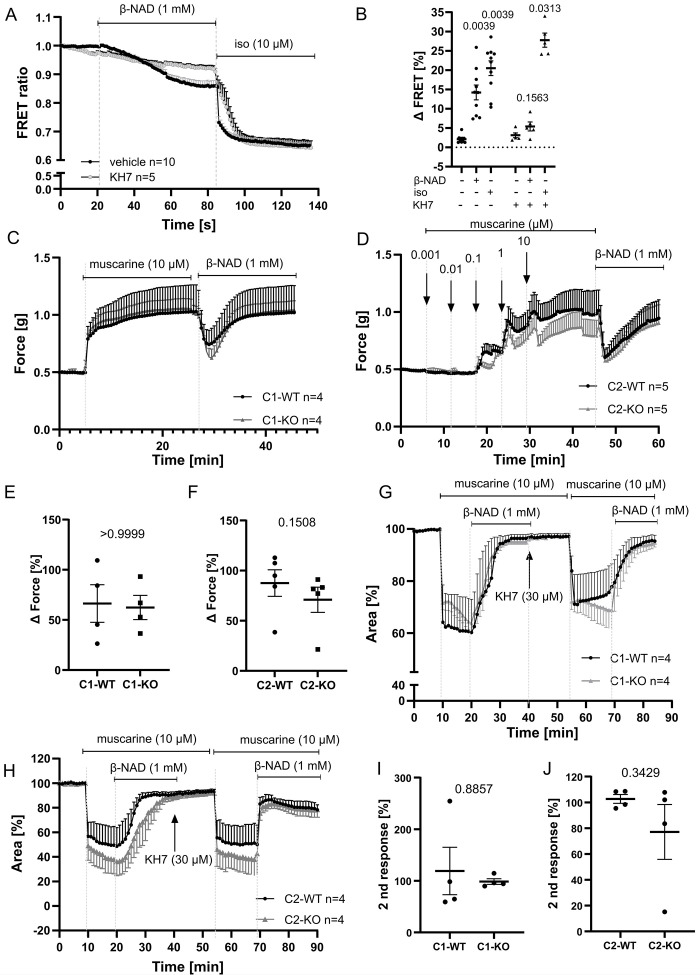
β-NAD increases intracellular cAMP concentration via soluble adenylyl cyclase, but this pathway is not essential for the relaxing effect. (A, B) Recording of intracellular cAMP concentration in HBSMC via FRET, with low FRET ratio indicating high cAMP concentration. β-NAD and isoproterenol cause a decrease in FRET ratio, reflecting rise in intracellular cAMP concentration. In the presence of KH7 (30 µM), a soluble adenylyl cyclase antagonist, the cAMP response to β-NAD was blocked, while the isoproterenol-induced cAMP increase remained unaffected. (A) FRET ratio over time and (B) ΔFRET ratio (%) represents the change in response to β-NAD and isoproterenol, measured in the presence and absence of KH-7. Within-group comparisons include the ΔFRET ratio before agonist addition versus after the addition of β-NAD or isoproterenol. n represents the number of cells analyzed, derived from three independent experiments. Error bars indicate mean ± SEM throughout. Statistical analysis was performed using the Wilcoxon signed-rank test (two-tailed) with Bonferroni correction for multiple comparisons. (C-J) Force recording from trachea in organ bath (C-F) and videomorphometric recording of luminal bronchial area in PCLS (G-J) in specimens taken from mice lacking the C1 (C, E, G, I) or C2 domain of soluble adenylyl cyclase (D, F, H, J). In both assays, β-NAD-induced relaxation of muscarine-precontracted airways was not significantly reduced in knockout mice compared to their respective wildtype controls. (G-J) In PCLS, KH7 (30 µM) has no significant effect upon muscarine-induced contraction and β-NAD-induced relaxation, both in knockout and in wild-type mice. (E, F, I, J) Scatterplots depict changes induced by β-NAD related to the preceding response to muscarine. (E, F) Scatterplots show the β-NAD-induced relaxation effect (%) relative to the muscarine response. (I, J) Scatterplot showing the maximum peak responses of the second stimulation (first response set as 100%) in the presence of KH7 within C1 and C2 knockout groups and their corresponding wild-type controls. The corresponding controls with the application of vehicle (DMSO) instead of KH7 are depicted in S7 Fig. Statistical analysis was performed using the Mann-Whitney test. Data are expressed as mean ± SEM. n refers to the number of animals.

Since the phosphodiesterase inhibitor rolipram enhanced β-NAD-induced tracheal and bronchial relaxation in murine airways (Fig 3D and 3E), we tested for an effect of β-NAD on intracellular cAMP concentration in human bronchial smooth muscle cells transfected with a cAMP-sensitive FRET sensor. β-NAD (1 mM) evoked a decrease in FRET ratio, indicative of an increase in intracellular cAMP concentration, that was further augmented by isoproterenol (10 µM), an activator of membrane-bound adenylate cyclase through β2-adrenoreceptor/G_s_-coupling (Fig 6A). This additive effect suggested an action of β-NAD on soluble adenylate cyclase (SAC). Accordingly, the SAC inhibitor KH7 (30 µM) blocked the effect of β-NAD while that of isoproterenol remained unaffected (Fig 6A and 6B).

Although this increase in intracellular cAMP through SAC activation offered a potential explanation of the β-NAD-induced airway relaxation, SAC inhibitor experiments, and the use of two different SAC knockout mouse strains did not support this hypothesis. KH7, a soluble adenylyl cyclase (sAC) inhibitor, did not significantly affect β-NAD-induced relaxation in either tracheal organ bath preparations or PCLS from murine airways pre-contracted with 10 µM muscarine. However, KH7-treated tissues showed slightly reduced muscarine-induced pre-contraction (S7 Fig), which may reflect off-target effects of the compound rather than selective sAC inhibition.

Similarly, β-NAD remained fully effective in tissues from mice lacking either the C1 or C2 domain of sAC (Figs 6C–6J and S7), further arguing against an essential role of sAC in this pathway.

To better understand the molecular components of cAMP signaling relevant to β-NAD-induced relaxation, we analyzed publicly available single-cell RNA sequencing datasets from mouse and human airway tissues. ADCY, PDE, and calcium signaling gene expression profiles were assessed in epithelial and smooth muscle cell populations (S8 Fig). Notably, Adcy10 (encoding sAC) was detected in most human airway cell types but showed low expression in smooth muscle cells (S9 Fig). In contrast, transmembrane adenylyl cyclases Adcy2, Adcy3, and Adcy9 were more strongly expressed—particularly in secretory and chondrocyte populations. Importantly, Pde4d was consistently expressed in both epithelial and smooth muscle cells across species, aligning with the observed effect of PDE4 inhibition.

Additional cAMP pathway components, including Ryr2, Itpr1, and PRKAR1A, were also broadly expressed in airway tissues, suggesting a functional role for cAMP-dependent signaling in airway tone regulation. Further inspection of smooth muscle–specific ACTA2 ⁺ populations confirmed minimal expression of Adcy10 and strong expression of transmembrane cyclases and Pde4d (S3 Table).

Together, these results support a model in which β-NAD elevates intracellular cAMP in part through sAC, but this mechanism is dispensable for its relaxing effect. Instead, β-NAD may act through transmembrane adenylyl cyclases and PDE4-sensitive cAMP pools to regulate airway smooth muscle tone.

## Discussion

This study identifies extracellular β-NAD as a direct bronchodilator acting on airway smooth muscle (ASM), as supported by cellular and tissue-level data.Notably, this effect operates through mechanisms that are principally different from the initially reported role as an inhibitory neurotransmitter in the intestine and urinary bladder. Smooth muscle preparations from these organs show spontaneous contractile activity, which is concentration-dependently suppressed by β-NAD [16,18,19,31,32]. In the original model, β-NAD exerts this effect through activating purinergic P2Y1 receptors, predominantly located on specialized interstitial cells rather than on the smooth muscle cells itself. In the intestine, these interstitial cells express platelet-derived growth factor-α and are interposed between inhibitory nerve fibers and smooth muscle cells. In contrast to the interstitial cells of Cajal (ICC), they do not serve as pacemakers, but rather cause muscle hyperpolarization through the sequence of events: P2Y1 receptor-mediated Ca^2+^-release from the endoplasmic reticulum, activation of small-conductance K^+^ (SK) channels with hyperpolarization of the interstitial cell, and transmission to the smooth muscle cell via gap junctions [31]. Clearly, this differs markedly from the mechanisms of the presently observed airway relaxation since P2Y inhibition (PPADS) and SK channel blockade (apamin) were ineffective, and we recorded depolarization rather than hyperpolarization from murine bronchial smooth muscle during β-NAD-induced relaxation. Further, the structural substrate, i.e., interstitial cells connected to smooth muscle cells with gap junctions, has not been observed in ASM [33,34].

This model of β-NAD acting as an inhibitory neurotransmitter has been questioned [35], and in an alternative scenario, it does not directly act upon intestinal smooth muscle or interstitial cells, but rather at prejunctional adenosine A_1_ receptors on nerve fibers [36]. *Per se*, this appeared unlikely to explain the β-NAD effect in the airways, because i) stimulation of adenosine A receptors causes bronchoconstriction and airway hyperresponsiveness rather than relaxation [37,38], and ii) inhibitory innervation of ASM is restricted to central airways and does not extend to smaller bronchi and bronchioli [39,40], in which we still observed the relaxant effect of β-NAD. Accordingly, β-NAD-induced airway relaxation was insensitive to the general adenosine receptor antagonist 8-(p-sulfophenyl) theophylline [41].

In isolated blood vessels, β-NAD has been reported to exert both relaxant and constrictor effects, depending on the vascular bed. Regardless of the direction of the response, these effects were consistently mediated via P2 purinergic or adenosine receptors [42]. In contrast, inhibition of these receptor families did not alter β-NAD-induced airway smooth muscle relaxation in the present study, indicating that previously proposed purinergic and adenosinergic mechanisms do not account for β-NAD’s effect in the airways.

By comparison, in vascular, gastrointestinal and urinary smooth muscles, β-NAD-induced relaxation has been repeatedly linked to P2Y₁ receptor signaling. For example, Mutafova-Yambolieva et al. demonstrated that β-NAD causes vasorelaxation in mesenteric arteries via P2Y₁ receptors, which was blocked by MRS2179 and PPADS [16]. Similarly, Alefishat et al. showed that MRS2179 antagonized β-NAD responses in bladder smooth muscle [42], and Goyal et al. confirmed the role of MRS2500 in blocking β-NAD-induced hyperpolarization and relaxation in mouse colon [43]. Wang et al. reported suppression of inhibitory junction potentials in guinea pig colon and human jejunum via adenosine A1 receptors, indicating a prejunctional neuromodulatory role [36].

In other studies, broad-spectrum purinergic antagonists such as suramin effectively blocked β-NAD responses in intestinal and urogenital tissues [19,44]. Breen et al. further identified β-NAD release from nerve terminals in the human bladder, reinforcing its role as a neurotransmitter [17].

Collectively, these studies underline the mechanistic divergence of β-NAD action between airway and other smooth muscles and emphasize that, unlike in gastrointestinal or urinary systems, β-NAD relaxation in ASM is independent of classical P2Y₁ or adenosine receptor pathways. To further explore possible signaling pathways, we examined whether β-NAD interferes with muscarinic acetylcholine receptor signaling, a prototypical bronchoconstrictor mechanism. However, neither general interference with Gs nor Gi proteins, using cholera toxin and pertussis toxin, respectively, affected β-NAD-induced relaxation.

At the cellular level, β-NAD evoked a rise in [Ca^2+^]_i_ in isolated murine and human ASM, which is uncommon for relaxing agents. Instead, calcium release from intracellular stores with subsequent increase in [Ca^2+^]_i_ of calcium oscillations is known as an essential step in canonical bronchoconstrictor pathways such as cholinergic bronchoconstriction [45,46]. Accordingly, [Ca^2+^]_i_ increase was also observed in the present study in murine bronchial ASM in PCLS stimulated with muscarine. Nonetheless, a paradoxical rise in [Ca^2+^]_i_ has also been reported concomitant to ASM relaxation induced by bitter compounds. It has been interpreted as a localized [Ca^2+^]_i_ response at the cell membrane, resulting in the opening of large-conductance Ca²^+^-activated K^+^ channels, thereby leading to ASM membrane hyperpolarization [47]. This model is still under debate. Specifically, it has been reported that bitter agonists evoke different [Ca^2+^]_i_ responses in rest and precontracted ASM, with a reversal of [Ca^2+^]_i_ increases or inhibition of calcium oscillations induced by cholinergic stimulation [45,46]. Similar paradoxical [Ca^2+^]_i_-increasing effects have been observed for isolated gallbladder smooth muscle cells with bitter agonists [48].

It remains to be determined whether β-NAD-induced [Ca^2+^]_i_ increase and membrane depolarization are mechanistically linked to relaxation or occur concomitantly without a causal relationship. It should also be noted that our measurements reflect global cytosolic [Ca²⁺]ᵢ and relative changes in overall membrane potential, whereas subplasmalemmal microdomains could not be resolved with the methods used.

In contrast to the seemingly paradoxical rise in [Ca^2+^]_i_, the observed increase in intracellular cAMP concentration matches with current models of smooth muscle relaxation. Cyclic AMP is generated from ATP by two types of adenylyl cyclases: the G protein-regulated transmembrane adenylyl cyclases and SAC at various intracellular locations [49,50]. Activation of membrane-bound adenylate cyclase through a G_s_ protein is the initial step in β2-adrenoreceptor-evoked bronchodilation [51]. β-NAD, in contrast, increased intracellular cAMP via SAC, as evidenced by selective inhibition by KH7 and additivity to β2-adrenoreceptor-induced increase in intracellular cAMP. In smooth muscle, SAC thus far has been linked to the induction of apoptosis in a PKA-dependent manner [52,53], but not to relaxation. Our present data also do not support such a relaxant role, since β-NAD-induced relaxation of precontracted airways was unaffected by inhibition of SAC (KH7) and of PKA (Rp-cAMP) and fully persisted in airways from mice with genetic deletion of either the C1 or C2 catalytic domain of SAC. Thus, even though β-NAD induced an increase in cAMP concentration, this did not translate into relaxation, probably because cAMP generation and signaling are compartmentalized within cells and its diffusion is limited, e.g., by cAMP degrading phosphodiesterases [54,55]. Accordingly, the inhibition of phosphodiesterase-4 (rolipram), expected to increase intracellular cAMP spread, augmented β-NAD-induced relaxation. In line with this assumption, the inhibition of phosphodiesterase-4 (PDE4) with rolipram, which promotes cAMP accumulation, significantly augmented β-NAD-induced relaxation. This finding is consistent with previous studies in guinea pig airway smooth muscle, where PDE4 inhibition enhanced β-adrenoceptor-mediated relaxation, particularly in tissues with distinct β-adrenoceptor subtype expression [56]. Although the primary receptors differ, both pathways converge on cAMP signaling, reinforcing the role of PDE4 in regulating airway tone across species. Our findings extend this concept to β-NAD, a non-adrenergic relaxant, and suggest that cAMP compartmentalization may limit its effect unless PDE4 activity is inhibited. Given that β-NAD-induced relaxation persisted in sAC knockout mice and was unaffected by the sAC inhibitor KH7, we did not further investigate the mechanism of sAC activation, although the role of soluble adenylyl cyclase remains unresolved. Transcriptomic analysis of both human and mouse airway smooth muscle cells revealed low expression of ADCY10 (sAC) relative to several transmembrane adenylyl cyclases, including ADCY2, ADCY3, and ADCY9. These observations point toward a dominant role of transmembrane ACs in mediating β-NAD-induced cAMP signaling. However, we cannot exclude the possibility that alternative sAC isoforms or context-dependent regulatory mechanisms contribute to the observed effects. Further studies are needed to fully delineate the source and regulation of cAMP production in this setting.

Importantly, since sAC is an intracellular enzyme, we could not identify transmembrane receptors or channels as direct or indirect β-NAD targets in the relaxant pathway. Thus, it has to be considered that β-NAD entered the cells and exerted its effect through intracellular targets. In cell culture, significant uptake of β-NAD, e.g., through connexin hemichannels, with a plethora of intracellular effects is reported at extracellular concentrations of 0.1 mM and higher [57,58]. This matches the concentration range in which we observed a relaxant effect, whereas β-NAD effects on purinergic receptors, the best-characterized targets of extracellular β-NAD, occur at lower concentrations down to 0.1 µM [16,42].

While the molecular pathway through which β-NAD acts on contracted airways remains to be elucidated, the present data identify it as a strong bronchodilator, which can fully counteract cholinergic bronchoconstriction in both murine and human airways. The clinical need for bronchodilation is mostly given in settings with concomitant inflammation, such as asthma and COPD. Intriguingly, β-NAD inhibits ATP-induced inflammasome activation [59,60] and attenuates neutrophil infiltration, capillary leak, and up-regulation of proinflammatory cytokine mRNA in lipopolysaccharide-induced acute lung injury in mice [11]. This has already led to the suggestion to explore its therapeutic potential in acute lung injury [11]. Exogenous β-NAD administration also counteracts oxidative stress and protects against myocardial ischemia/reperfusion injury in a rat model [61]. These anti-inflammatory and antioxidant effects could add value to β-NAD as a bronchodilator for use in COPD, asthma, or acute respiratory distress syndrome (ARDS). Furthermore, phosphodiesterase-4 inhibition, a therapeutic strategy that is already an add-on treatment for patients with severe COPD associated with bronchitis and a history of frequent exacerbations [62–64], enhanced the β-NAD effect and might be considered a supportive principle.

## Supporting information

S1 Fig β-NAD does not interfere with the contraction phase. (A) Videomorphometric recordings from murine PCLS showing contraction responses to KCl. PCLS were first contracted with KCl, followed by treatment with varying doses of β-NAD, and then re-challenged with KCl. (B) Force recordings from tracheal segments in an organ bath. Tracheal tissue was first exposed to 5 mM β-NAD, followed by muscarine-induced contraction. In both experiments, the contractile responses to KCl (in PCLS) and muscarine (in the trachea) remained unchanged after β-NAD treatment. N indicates the number of animals, or in the case of PCLS, the number of airways studied, followed by the number of animals used for PCLS preparation, presented as airway/mice. Data are expressed as mean ± SEM.
(TIF)

S2 Fig β-NAD induces faster and equally effective relaxation of muscarine-precontracted bronchi compared to salbutamol. (A) Videomorphometric recordings of the luminal bronchial area in PCLS demonstrate that muscarine-induced bronchoconstriction is fully reversed by β-NAD (1 mM; solid line) and salbutamol (100 µM; dashed line), with β-NAD achieving maximal relaxation more rapidly. N indicates the number of airways studied, followed by the number of animals used for PCLS preparation, presented as airway/mice. Data are expressed as mean ± SEM. Statistical analysis (Mann-Whitney U test) reveals that β-NAD induced significantly stronger relaxation at 25 and 35 minutes, while both β-NAD and salbutamol reached comparable maximal relaxation by 40 minutes. (B) Representative PCLS images illustrate the bronchial area at specific time points (5, 15, and 35 minutes) during the experiment.
(TIF)

S3 Fig ATP-induced bronchorelaxation is inhibited by purinergic receptor blockers. Videomorphometric recordings from murine PCLS demonstrate ATP-induced bronchorelaxation. Muscarine-induced constriction (10 µM) is normalized to 100%. The dashed line represents data from PCLS pretreated with a purinergic receptor inhibitor cocktail (P-Block) comprising suramin (100 µM), pyridoxalphosphate-6-azophenyl-2’,4’-disulfonic acid (30 µM), and MRS2179 (10 µM). N indicates the number of animals, or in the case of PCLS, the number of airways studied, followed by the number of animals used for PCLS preparation, presented as airway/mice. Data are expressed as mean ± SEM. The scatter plot compares the maximum relaxation evoked by ATP in the absence and presence of the P-Block cocktail. Data are presented as means ± SEM, with statistical significance assessed using the Mann-Whitney U test.
(TIF)

S4 Fig CTX reduces salbutamol-induced relaxation of tracheal segments. Force recordings from tracheal segments in an organ bath show that muscarine-induced constriction (10 µM) is set as 100%. Pretreatment with cholera toxin (CTX, 2 ng/ml) significantly attenuated salbutamol-induced relaxation of the tracheal segments. The scatterplot depicts β-NAD-induced relaxation as a percentage of the muscarine response in the absence (vehicle) and presence of CTX. N indicates the number of animals. Data are presented as means ± SEM, and statistical analysis was performed using the Mann-Whitney test.
(TIF)

S5 Fig Effect of inhibitors upon muscarine-induced constriction of tracheal segments. Muscarine-induced contraction data are normalized to the respective vehicle control for each experimental setup, with the vehicle response defined as 100%. The scatterplot displays the percentage of muscarine-induced contraction, with p-values shown above the data points (compared to the respective vehicle control, Kruskal-Walli’s test followed by Dunn’s multiple comparisons test). Whiskers represent the mean ± SEM. N indicates the number of animals. Substances tested include vehicle controls (0.03% DMSO, PBS, 0.01% DMSO, or 0.02% DMSO), purinergic receptor inhibitors (8-sPT, 10 µM; apamin, 10 µM; and P-Block comprising suramin, 100 µM, and PPADS, 30 µM), Rp-cAMP (100 µM), U-73122 (10 µM), cholera toxin (CTX, 2 ng/ml), pertussis toxin (PTX, 5 ng/ml), L-NAME (100 µM), rolipram (100 µM), and FPL64176 (10 µM). Among the tested substances, 8-sPT, apamin and Rp-cAMP significantly increased muscarine-induced contraction, while U-73122 inhibited muscarine responses.
(TIF)

S6 Fig β-NAD does not elevate intracellular calcium levels in M3WT4 cells. The Fura-2 340/380 fluorescence ratio of M3 muscarinic acetylcholine receptor-expressing M3WT4 cells is shown. The addition of β-NAD did not increase intracellular calcium levels, as indicated by the unchanged fluorescence ratio. Acetylcholine, used as a positive control at the end of the experiment, induced a robust increase, confirming the functionality of the receptor and the responsiveness of the assay. N indicates the number of cells.
(TIF)

S7 Fig Inhibition or deletion of SAC does not impair the relaxant effect of β-NAD. (A) Force recordings from tracheal segments in an organ bath preincubated with KH7 (30 µM; gray line), a SAC inhibitor dissolved in DMSO, or vehicle control (DMSO; final concentration: 0.003%; black line). β-NAD induced relaxation of muscarine-precontracted trachea in both KH7-treated and vehicle-treated tissues, with no significant difference observed between the two groups. (B, C) Videomorphometric recordings of the bronchial luminal area in PCLS, showing vehicle controls corresponding to Fig 6G and 6H. Two cycles of muscarine-induced contraction and β-NAD-induced relaxation were performed with vehicle (DMSO, 0.03%) applied between cycles. No significant differences in β-NAD-induced relaxation were observed between SAC C1 (B) or SAC C2 (C) knockout (KO) mice and their respective wild-type (WT) controls, nor were differences noted due to vehicle application. Scatterplot showing the maximum peak responses of the second stimulation (first response set as 100%) in the presence of the vehicle (DMSO treatment) within C1 and C2 knockout groups and their corresponding wild-type controls (B, C). Data are shown as means ± SEM. Statistical analysis was performed using the Mann-Whitney test.
(TIF)

S8 Fig Expression of ADCY, PDE, and calcium signaling genes in mouse and human airway epithelial and smooth muscle cells. Single-cell RNA sequencing analysis of airway tissues from mice and human (datasets: GSE136831, GSE134174, GSE244215). (A) Mouse epithelial cells (EPCAM⁺). (B) Mouse airway smooth muscle cells (ACTA2⁺). (C) Human epithelial cells (EPCAM⁺). (D) Human airway smooth muscle cells (ACTA2⁺). Data are shown as UMI counts per cell. ADCY2, ADCY3, and ADCY9 are enriched in both epithelial and smooth muscle compartments. ADCY10 shows low expression in smooth muscle cells. PDE4B, PDE4D, RYR2, ITPR1, and PRKAR1A are expressed in both cell types, whereas ITPR3 is largely confined to epithelial cells. Gene symbols are presented in human nomenclature; corresponding mouse orthologs follow standard capitalization rules.
(TIF)

S9 Fig Expression of adenylyl cyclase and phosphodiesterase genes in human airway cell and nuclear preparations. Single-cell and single-nucleus RNA-sequencing data from all major human airway cell types (datasets: GSE136831, GSE134174, GSE244215). Data are shown as UMI counts per cell. ADCY10 is broadly expressed across the dataset, while ADCY2 shows high expression in secretory goblet and chondrocyte cells. PDE4D, a phosphodiesterase relevant to β-NAD signaling, is widely expressed in both epithelial and mesenchymal compartments, supporting a role for phosphodiesterase regulation in airway smooth muscle relaxation.
(TIF)

S1 Table List of reagents and resources used in this study. (DOCX)

S2 Table Characteristics of drugs used in organ bath experiments to elucidate signaling pathways. (DOCX)

S3 Table In silico expression analysis of cAMP signaling pathway genes in human and mouse airway cell types based on single-cell RNA sequencing. Expression of key cAMP signaling pathway genes in human and mouse airway epithelial (EPCAM⁺), smooth muscle (ACTA2⁺), and ADCY10 ⁺ cells was analyzed using single-cell RNA-seq datasets GSE136831, GSE134174, and GSE244215. Values represent the total number of cells expressing genes of interest with a UMI score > 1.0. The data highlight differential expression patterns of adenylyl cyclase isoforms (ADCY2/3/9 vs. ADCY10), phosphodiesterases (PDE4B/D), and cAMP effectors (PRKAR1A, RYR2, ITPR1) across species and cell types.
(DOCX)

## References

[1] Hassoun D, Rose L, Blanc F-X, Magnan A, Loirand G, Sauzeau V. Bronchial smooth muscle cell in asthma: where does it fit? BMJ Open Respir Res. 2022;9(1):e001351. doi: 10.1136/bmjresp-2022-001351 36109087 PMC9478857

[2] Boers E, Barrett M, Su JG, Benjafield AV, Sinha S, Kaye L, et al. Global burden of chronic obstructive pulmonary disease through 2050. JAMA Netw Open. 2023;6(12):e2346598. doi: 10.1001/jamanetworkopen.2023.46598 38060225 PMC10704283

[3] GBD 2019 Chronic Respiratory Diseases Collaborators. Global burden of chronic respiratory diseases and risk factors, 1990-2019: an update from the Global Burden of Disease Study 2019. EClinicalMedicine. 2023;59:101936. doi: 10.1016/j.eclinm.2023.101936 37229504 PMC7614570

[4] Kume H. Role of airway smooth muscle in inflammation related to asthma and COPD. Adv Exp Med Biol. 2021;1303:139–72. doi: 10.1007/978-3-030-63046-1_9 33788192

[5] Camoretti-Mercado B, Lockey RF. Airway smooth muscle pathophysiology in asthma. J Allergy Clin Immunol. 2021;147(6):1983–95. doi: 10.1016/j.jaci.2021.03.035 34092351

[6] Pera T, Penn RB. Crosstalk between beta-2-adrenoceptor and muscarinic acetylcholine receptors in the airway. Curr Opin Pharmacol. 2014;16:72–81. doi: 10.1016/j.coph.2014.03.005 24747364 PMC4096844

[7] Gosens R, Zaagsma J, Meurs H, Halayko AJ. Muscarinic receptor signaling in the pathophysiology of asthma and COPD. Respir Res. 2006;7(1):73. doi: 10.1186/1465-9921-7-73 16684353 PMC1479816

[8] Nakamura M, Bhatnagar A, Sadoshima J. Overview of pyridine nucleotides review series. Circ Res. 2012;111(5):604–10. doi: 10.1161/CIRCRESAHA.111.247924 22904040 PMC3523884

[9] Fang J, Chen W, Hou P, Liu Z, Zuo M, Liu S, et al. NAD+ metabolism-based immunoregulation and therapeutic potential. Cell Biosci. 2023;13(1):81. doi: 10.1186/s13578-023-01031-5 37165408 PMC10171153

[10] Okabe K, Yaku K, Tobe K, Nakagawa T. Implications of altered NAD metabolism in metabolic disorders. J Biomed Sci. 2019;26(1):34. doi: 10.1186/s12929-019-0527-8 31078136 PMC6511662

[11] Umapathy NS, Gonzales J, Fulzele S, Kim K, Lucas R, Verin AD. β-Nicotinamide adenine dinucleotide attenuates lipopolysaccharide-induced inflammatory effects in a murine model of acute lung injury. Exp Lung Res. 2012;38(5):223–32. doi: 10.3109/01902148.2012.673049 22563684 PMC3678723

[12] Ehrsam JP, Chen J, Haberecker M, Arni S, Inci I. Effect of β-Nicotinamide adenine dinucleotide on acute allograft rejection after rat lung transplantation. Transplant Direct. 2023;9(9):e1516. doi: 10.1097/TXD.0000000000001516 37575952 PMC10414733

[13] Elkhal A, Rodriguez Cetina Biefer H, Heinbokel T, Uehara H, Quante M, Seyda M, et al. NAD(+) regulates Treg cell fate and promotes allograft survival via a systemic IL-10 production that is CD4(+) CD25(+) Foxp3(+) T cells independent. Sci Rep. 2016;6:22325. doi: 10.1038/srep22325 26928119 PMC4772111

[14] Tullius SG, Biefer HRC, Li S, Trachtenberg AJ, Edtinger K, Quante M, et al. NAD+ protects against EAE by regulating CD4+ T-cell differentiation. Nat Commun. 2014;5:5101. doi: 10.1038/ncomms6101 25290058 PMC4205890

[15] Rodriguez Cetina Biefer H, Heinbokel T, Uehara H, Camacho V, Minami K, Nian Y, et al. Mast cells regulate CD4+ T-cell differentiation in the absence of antigen presentation. J Allergy Clin Immunol. 2018;142(6):1894-1908.e7. doi: 10.1016/j.jaci.2018.01.038 29470999 PMC6454881

[16] Mutafova-Yambolieva VN, Hwang SJ, Hao X, Chen H, Zhu MX, Wood JD, et al. Beta-nicotinamide adenine dinucleotide is an inhibitory neurotransmitter in visceral smooth muscle. Proc Natl Acad Sci U S A. 2007;104(41):16359–64. doi: 10.1073/pnas.0705510104 17913880 PMC2042211

[17] Breen LT, Smyth LM, Yamboliev IA, Mutafova-Yambolieva VN. beta-NAD is a novel nucleotide released on stimulation of nerve terminals in human urinary bladder detrusor muscle. Am J Physiol Renal Physiol. 2006;290(2):F486-95. doi: 10.1152/ajprenal.00314.2005 16189287

[18] Durnin L, Sanders KM, Mutafova-Yambolieva VN. Differential release of β-NAD(+) and ATP upon activation of enteric motor neurons in primate and murine colons. Neurogastroenterol Motil. 2013;25(3):e194-204. doi: 10.1111/nmo.12069 23279315 PMC3578016

[19] Hwang SJ, Durnin L, Dwyer L, Rhee P-L, Ward SM, Koh SD, et al. β-nicotinamide adenine dinucleotide is an enteric inhibitory neurotransmitter in human and nonhuman primate colons. Gastroenterology. 2011;140(2):608-617.e6. doi: 10.1053/j.gastro.2010.09.039 20875415 PMC3031738

[20] Esposito G, Jaiswal BS, Xie F, Krajnc-Franken MAM, Robben TJAA, Strik AM, et al. Mice deficient for soluble adenylyl cyclase are infertile because of a severe sperm-motility defect. Proc Natl Acad Sci U S A. 2004;101(9):2993–8. doi: 10.1073/pnas.0400050101 14976244 PMC365733

[21] Chen J, Martinez J, Milner TA, Buck J, Levin LR. Neuronal expression of soluble adenylyl cyclase in the mammalian brain. Brain Res. 2013;1518:1–8. doi: 10.1016/j.brainres.2013.04.027 23611875 PMC3679342

[22] Martin C, Uhlig S, Ullrich V. Cytokine-induced bronchoconstriction in precision-cut lung slices is dependent upon cyclooxygenase-2 and thromboxane receptor activation. Am J Respir Cell Mol Biol. 2001;24(2):139–45. doi: 10.1165/ajrcmb.24.2.3545 11159047

[23] Paddenberg R, König P, Faulhammer P, Goldenberg A, Pfeil U, Kummer W. Hypoxic vasoconstriction of partial muscular intra-acinar pulmonary arteries in murine precision cut lung slices. Respir Res. 2006;7(1):93. doi: 10.1186/1465-9921-7-93 16808843 PMC1524949

[24] Keshavarz M, Schwarz H, Hartmann P, Wiegand S, Skill M, Althaus M, et al. Caveolin-1: functional insights into its role in muscarine- and serotonin-induced smooth muscle constriction in murine airways. Front Physiol. 2017;8:295. doi: 10.3389/fphys.2017.00295 28555112 PMC5430063

[25] Nassenstein C, Wiegand S, Lips KS, Li G, Klein J, Kummer W. Cholinergic activation of the murine trachealis muscle via non-vesicular acetylcholine release involving low-affinity choline transporters. Int Immunopharmacol. 2015;29(1):173–80. doi: 10.1016/j.intimp.2015.08.007 26278668

[26] Keshavarz M, Skill M, Hollenhorst MI, Maxeiner S, Walecki M, Pfeil U, et al. Caveolin-3 differentially orchestrates cholinergic and serotonergic constriction of murine airways. Sci Rep. 2018;8(1):7508. doi: 10.1038/s41598-018-25445-1 29760450 PMC5951923

[27] Madissoon E, Oliver AJ, Kleshchevnikov V, Wilbrey-Clark A, Polanski K, Richoz N, et al. A spatially resolved atlas of the human lung characterizes a gland-associated immune niche. Nat Genet. 2023;55(1):66–77. doi: 10.1038/s41588-022-01243-4 36543915 PMC9839452

[28] Lin B, Shah VS, Chernoff C, Sun J, Shipkovenska GG, Vinarsky V, et al. Airway hillocks are injury-resistant reservoirs of unique plastic stem cells. Nature. 2024;629(8013):869–77. doi: 10.1038/s41586-024-07377-1 38693267 PMC11890216

[29] Wolf FA, Angerer P, Theis FJ. SCANPY: large-scale single-cell gene expression data analysis. Genome Biol. 2018;19(1):15. doi: 10.1186/s13059-017-1382-0 29409532 PMC5802054

[30] Ellis JL, Undem BJ. Non-adrenergic, non-cholinergic contractions in the electrically field stimulated guinea-pig trachea. Br J Pharmacol. 1990;101(4):875–80. doi: 10.1111/j.1476-5381.1990.tb14174.x 2085710 PMC1917838

[31] Mutafova-Yambolieva VN, Durnin L. The purinergic neurotransmitter revisited: a single substance or multiple players? Pharmacol Ther. 2014;144(2):162–91. doi: 10.1016/j.pharmthera.2014.05.012 24887688 PMC4185222

[32] Sanders KM. Enteric inhibitory neurotransmission, starting down under. Adv Exp Med Biol. 2016;891:21–9. doi: 10.1007/978-3-319-27592-5_3 27379631 PMC8325941

[33] Gabella G. Ultrastructure of the tracheal muscle in developing, adult and ageing guinea-pigs. Anat Embryol (Berl). 1991;183(1):71–9. doi: 10.1007/BF00185837 2053711

[34] Kuo K-H, Dai J, Seow CY, Lee C-H, van Breemen C. Relationship between asynchronous Ca2+ waves and force development in intact smooth muscle bundles of the porcine trachea. Am J Physiol Lung Cell Mol Physiol. 2003;285(6):L1345-53. doi: 10.1152/ajplung.00043.2003 12936908

[35] Goyal RK. Evidence for β-nicotinamide adenine dinucleotide as a purinergic, inhibitory neurotransmitter in doubt. Gastroenterology. 2011;141(4):e27; author reply e27-8. doi: 10.1053/j.gastro.2011.07.047 21888906

[36] Wang G-D, Wang X-Y, Liu S, Xia Y, Zou F, Qu M, et al. β-Nicotinamide adenine dinucleotide acts at prejunctional adenosine A1 receptors to suppress inhibitory musculomotor neurotransmission in guinea pig colon and human jejunum. Am J Physiol Gastrointest Liver Physiol. 2015;308(11):G955-63. doi: 10.1152/ajpgi.00430.2014 25813057 PMC4451321

[37] Ponnoth DS, Nadeem A, Tilley S, Mustafa SJ. Involvement of A1 adenosine receptors in altered vascular responses and inflammation in an allergic mouse model of asthma. Am J Physiol Heart Circ Physiol. 2010;299(1):H81-7. doi: 10.1152/ajpheart.01090.2009 20400685 PMC2904134

[38] Walaschewski R, Begrow F, Verspohl EJ. Impact and benefit of A(2B)-adenosine receptor agonists for the respiratory tract: mucociliary clearance, ciliary beat frequency, trachea muscle tonus and cytokine release. J Pharm Pharmacol. 2013;65(1):123–32. doi: 10.1111/j.2042-7158.2012.01580.x 23215695

[39] Fischer A, Hoffmann B. Nitric oxide synthase in neurons and nerve fibers of lower airways and in vagal sensory ganglia of man. Correlation with neuropeptides. Am J Respir Crit Care Med. 1996;154(1):209–16. doi: 10.1164/ajrccm.154.1.8680682 8680682

[40] Balentova S, Conwell S, Myers AC. Neurotransmitters in parasympathetic ganglionic neurons and nerves in mouse lower airway smooth muscle. Respir Physiol Neurobiol. 2013;189(1):195–202. doi: 10.1016/j.resp.2013.07.006 23891709

[41] Deshpande DA, Guedes AGP, Lund FE, Subramanian S, Walseth TF, Kannan MS. CD38 in the pathogenesis of allergic airway disease: Potential therapeutic targets. Pharmacol Ther. 2017;172:116–26. doi: 10.1016/j.pharmthera.2016.12.002 27939939 PMC5346344

[42] Alefishat E, Alexander SPH, Ralevic V. Effects of NAD at purine receptors in isolated blood vessels. Purinergic Signal. 2015;11(1):47–57. doi: 10.1007/s11302-014-9428-1 25315718 PMC4336311

[43] Goyal RK, Sullivan MP, Chaudhury A. Progress in understanding of inhibitory purinergic neuromuscular transmission in the gut. Neurogastroenterol Motil. 2013;25(3):203–7. doi: 10.1111/nmo.12090 23414428 PMC8630810

[44] Shiina T, Suzuki Y, Horii K, Sawamura T, Yuki N, Horii Y, et al. Purinergic inhibitory regulation of esophageal smooth muscle is mediated by P2Y receptors and ATP-dependent potassium channels in rats. J Physiol Sci. 2024;74(1):26. doi: 10.1186/s12576-024-00916-5 38654149 PMC11036717

[45] Zhang C-H, Lifshitz LM, Uy KF, Ikebe M, Fogarty KE, ZhuGe R. The cellular and molecular basis of bitter tastant-induced bronchodilation. PLoS Biol. 2013;11(3):e1001501. doi: 10.1371/journal.pbio.1001501 23472053 PMC3589262

[46] Tan X, Sanderson MJ. Bitter tasting compounds dilate airways by inhibiting airway smooth muscle calcium oscillations and calcium sensitivity. Br J Pharmacol. 2014;171(3):646–62. doi: 10.1111/bph.12460 24117140 PMC3969078

[47] Deshpande DA, Wang WCH, McIlmoyle EL, Robinett KS, Schillinger RM, An SS, et al. Bitter taste receptors on airway smooth muscle bronchodilate by localized calcium signaling and reverse obstruction. Nat Med. 2010;16(11):1299–304. doi: 10.1038/nm.2237 20972434 PMC3066567

[48] Keshavarz M, Ruppert A-L, Meiners M, Poharkar K, Liu S, Mahmoud W, et al. Bitter tastants relax the mouse gallbladder smooth muscle independent of signaling through tuft cells and bitter taste receptors. Sci Rep. 2024;14(1):18447. doi: 10.1038/s41598-024-69287-6 39117690 PMC11310472

[49] Bitterman JL, Ramos-Espiritu L, Diaz A, Levin LR, Buck J. Pharmacological distinction between soluble and transmembrane adenylyl cyclases. J Pharmacol Exp Ther. 2013;347(3):589–98. doi: 10.1124/jpet.113.208496 24091307 PMC3836311

[50] Wiggins SV, Steegborn C, Levin LR, Buck J. Pharmacological modulation of the CO2/HCO3-/pH-, calcium-, and ATP-sensing soluble adenylyl cyclase. Pharmacol Ther. 2018;190:173–86. doi: 10.1016/j.pharmthera.2018.05.008 29807057 PMC6484840

[51] Johnson M. The beta-adrenoceptor. Am J Respir Crit Care Med. 1998;158(5 Pt 3):S146-53. doi: 10.1164/ajrccm.158.supplement_2.13tac110 9817738

[52] Appukuttan A, Kasseckert SA, Kumar S, Reusch HP, Ladilov Y. Oxysterol-induced apoptosis of smooth muscle cells is under the control of a soluble adenylyl cyclase. Cardiovasc Res. 2013;99(4):734–42. doi: 10.1093/cvr/cvt137 23729662

[53] Kumar S, Appukuttan A, Maghnouj A, Hahn S, Peter Reusch H, Ladilov Y. Suppression of soluble adenylyl cyclase protects smooth muscle cells against oxidative stress-induced apoptosis. Apoptosis. 2014;19(7):1069–79. doi: 10.1007/s10495-014-0989-9 24781801

[54] Johnstone TB, Agarwal SR, Harvey RD, Ostrom RS. cAMP signaling compartmentation: adenylyl cyclases as anchors of dynamic signaling complexes. Mol Pharmacol. 2018;93(4):270–6. doi: 10.1124/mol.117.110825 29217670 PMC5820540

[55] Pozdniakova S, Ladilov Y. Functional significance of the Adcy10-dependent intracellular cAMP compartments. J Cardiovasc Dev Dis. 2018;5(2):29. doi: 10.3390/jcdd5020029 29751653 PMC6023465

[56] Tomkinson A, Karlsson JA, Raeburn D. Comparison of the effects of selective inhibitors of phosphodiesterase types III and IV in airway smooth muscle with differing beta-adrenoceptor subtypes. Br J Pharmacol. 1993;108(1):57–61. doi: 10.1111/j.1476-5381.1993.tb13439.x 8428213 PMC1907714

[57] Pittelli M, Felici R, Pitozzi V, Giovannelli L, Bigagli E, Cialdai F, et al. Pharmacological effects of exogenous NAD on mitochondrial bioenergetics, DNA repair, and apoptosis. Mol Pharmacol. 2011;80(6):1136–46. doi: 10.1124/mol.111.073916 21917911

[58] Okuda H, Nishida K, Higashi Y, Nagasawa K. NAD(+) influx through connexin hemichannels prevents poly(ADP-ribose) polymerase-mediated astrocyte death. Life Sci. 2013;92(13):808–14. doi: 10.1016/j.lfs.2013.02.010 23454167

[59] Hiller SD, Heldmann S, Richter K, Jurastow I, Küllmar M, Hecker A, et al. β-nicotinamide adenine dinucleotide (β-NAD) inhibits ATP-dependent IL-1β release from human monocytic cells. Int J Mol Sci. 2018;19(4):1126. doi: 10.3390/ijms19041126 29642561 PMC5979475

[60] Misawa T, Takahama M, Kozaki T, Lee H, Zou J, Saitoh T, et al. Microtubule-driven spatial arrangement of mitochondria promotes activation of the NLRP3 inflammasome. Nat Immunol. 2013;14(5):454–60. doi: 10.1038/ni.2550 23502856

[61] Zhang Y, Wang B, Fu X, Guan S, Han W, Zhang J, et al. Exogenous NAD(+) administration significantly protects against myocardial ischemia/reperfusion injury in rat model. Am J Transl Res. 2016;8(8):3342–50. 27648125 PMC5009387

[62] Chong J, Leung B, Poole P. Phosphodiesterase 4 inhibitors for chronic obstructive pulmonary disease. Cochrane Database Syst Rev. 2017;9(9):CD002309. doi: 10.1002/14651858.CD002309.pub5 28922692 PMC6483682

[63] Zuo H, Cattani-Cavalieri I, Musheshe N, Nikolaev VO, Schmidt M. Phosphodiesterases as therapeutic targets for respiratory diseases. Pharmacol Ther. 2019;197:225–42. doi: 10.1016/j.pharmthera.2019.02.002 30759374

[64] Kumar R, Khan MI, Panwar A, Vashist B, Rai SK, Kumar A. PDE4 inhibitors and their potential combinations for the treatment of chronic obstructive pulmonary disease: a narrative review. Open Respir Med J. 2024;18:e18743064340418. doi: 10.2174/0118743064340418241021095046 39839967 PMC11748061

