# Supplementary figures and images for: β-Nicotinamide adenine dinucleotide (β-NAD) acts as a bronchodilator
Source: PLoS One. 2025 Oct 14;20(10):e0334491. doi: 10.1371/journal.pone.0334491 (PMC12520353; doi:10.1371/journal.pone.0334491)

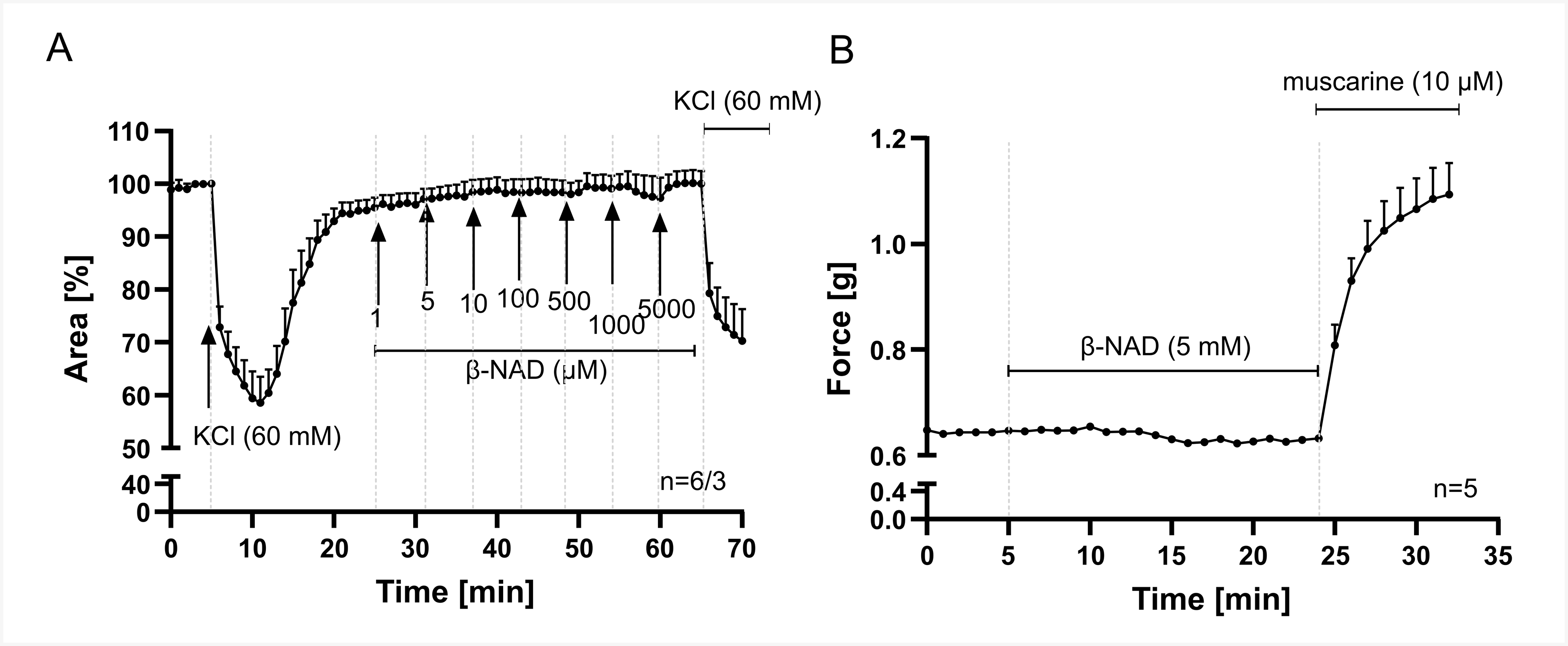

Supplement: S1 Fig — (A) Videomorphometric recordings from murine PCLS showing contraction responses to KCl. PCLS were first contracted with KCl, followed by treatment with varying doses of β-NAD, and then re-challenged with KCl. (B) Force recordings from tracheal segments in an organ bath. Tracheal tissue was first exposed to 5 mM β-NAD, followed by muscarine-induced contraction. In both experiments, the contractile responses to KCl (in PCLS) and muscarine (in the trachea) remained unchanged after β-NAD treatment. N indicates the number of animals, or in the case of PCLS, the number of airways studied, followed by the number of animals used for PCLS preparation, presented as airway/mice. Data are expressed as mean ± SEM. (TIF) [file pone.0334491.s001.tif]

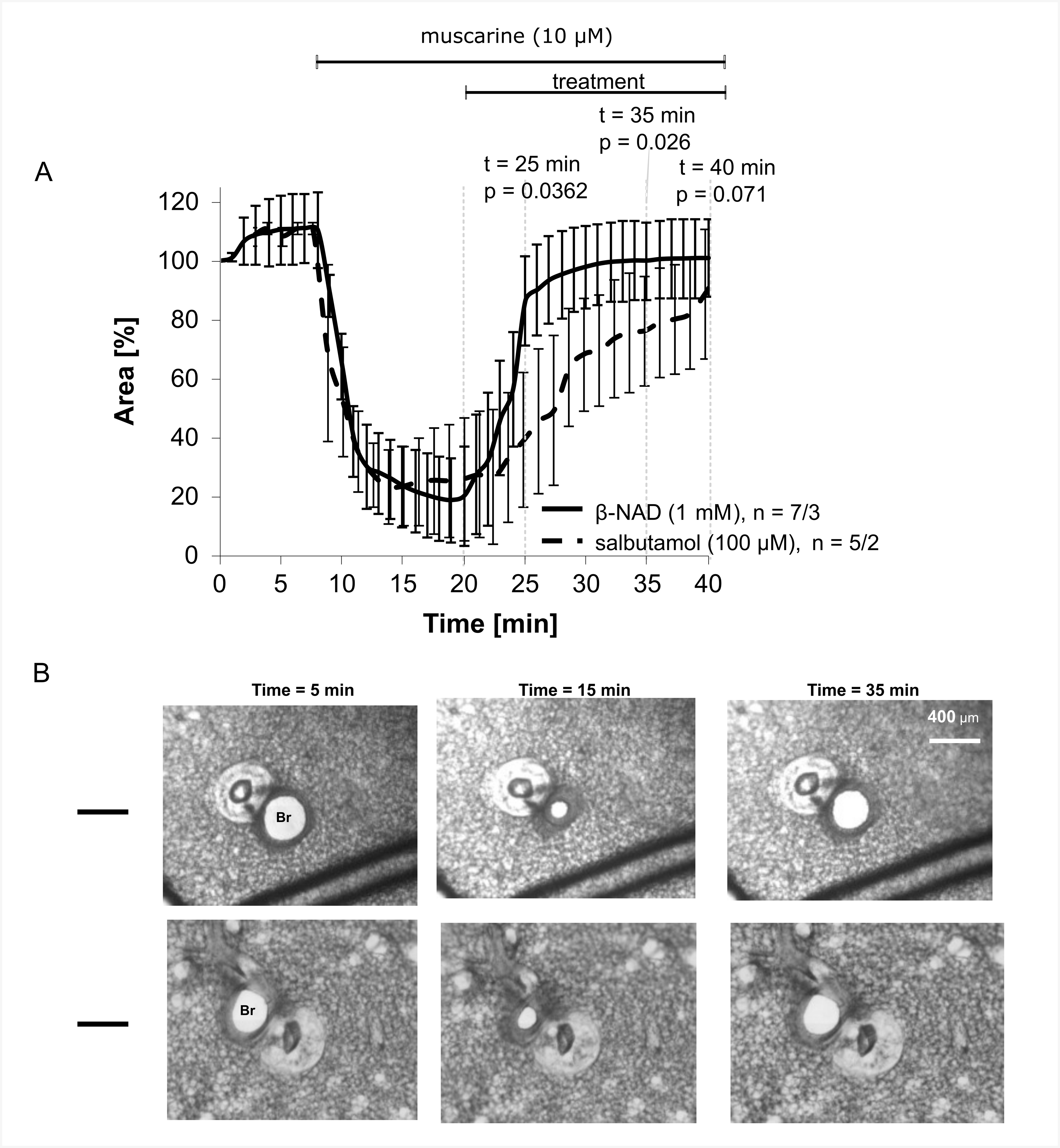

Supplement: S2 Fig — (A) Videomorphometric recordings of the luminal bronchial area in PCLS demonstrate that muscarine-induced bronchoconstriction is fully reversed by β-NAD (1 mM; solid line) and salbutamol (100 µM; dashed line), with β-NAD achieving maximal relaxation more rapidly. N indicates the number of airways studied, followed by the number of animals used for PCLS preparation, presented as airway/mice. Data are expressed as mean ± SEM. Statistical analysis (Mann-Whitney U test) reveals that β-NAD induced significantly stronger relaxation at 25 and 35 minutes, while both β-NAD and salbutamol reached comparable maximal relaxation by 40 minutes. (B) Representative PCLS images illustrate the bronchial area at specific time points (5, 15, and 35 minutes) during the experiment. (TIF) [file pone.0334491.s002.tif]

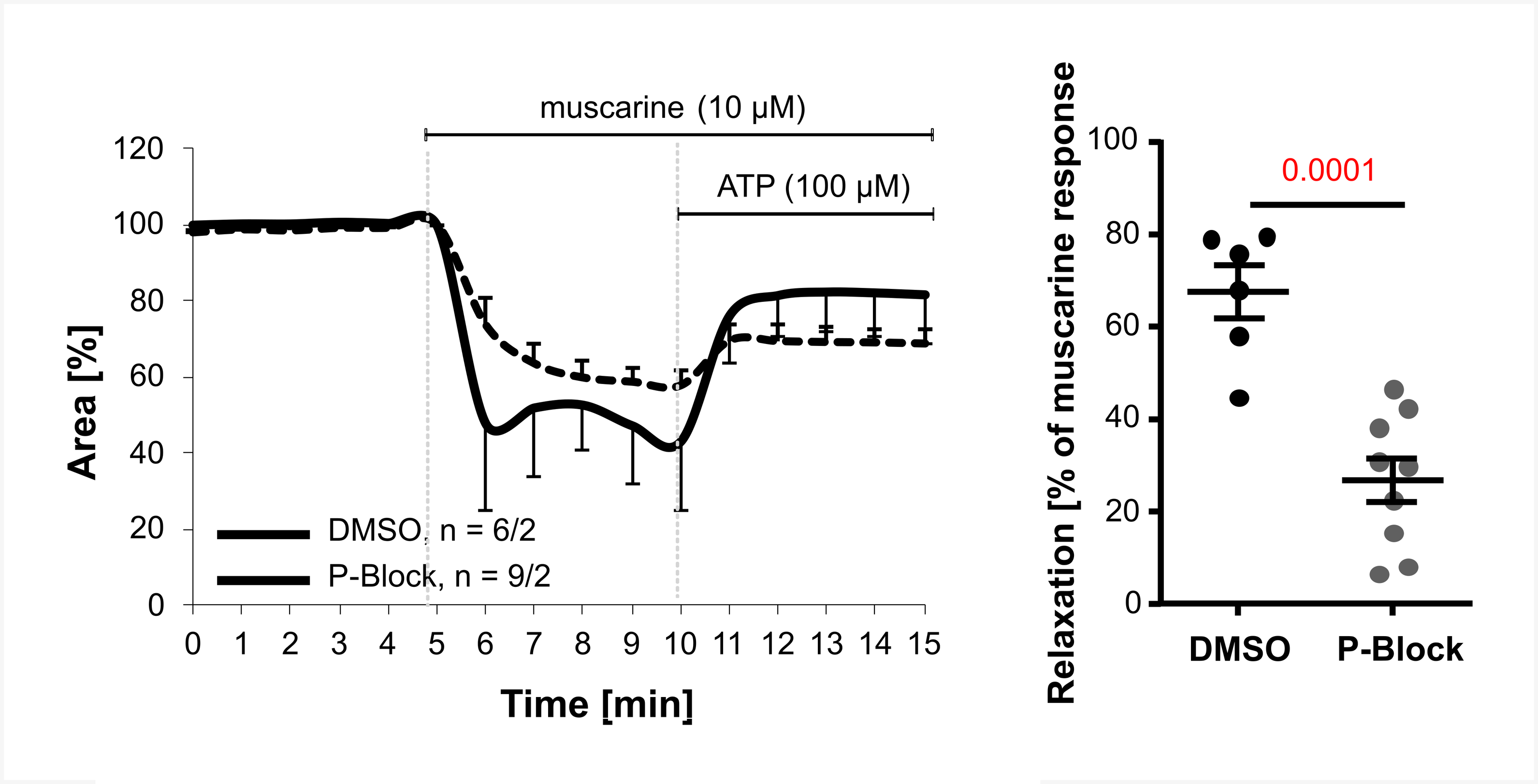

Supplement: S3 Fig — Videomorphometric recordings from murine PCLS demonstrate ATP-induced bronchorelaxation. Muscarine-induced constriction (10 µM) is normalized to 100%. The dashed line represents data from PCLS pretreated with a purinergic receptor inhibitor cocktail (P-Block) comprising suramin (100 µM), pyridoxalphosphate-6-azophenyl-2’,4’-disulfonic acid (30 µM), and MRS2179 (10 µM). N indicates the number of animals, or in the case of PCLS, the number of airways studied, followed by the number of animals used for PCLS preparation, presented as airway/mice. Data are expressed as mean ± SEM. The scatter plot compares the maximum relaxation evoked by ATP in the absence and presence of the P-Block cocktail. Data are presented as means ± SEM, with statistical significance assessed using the Mann-Whitney U test. (TIF) [file pone.0334491.s003.tif]

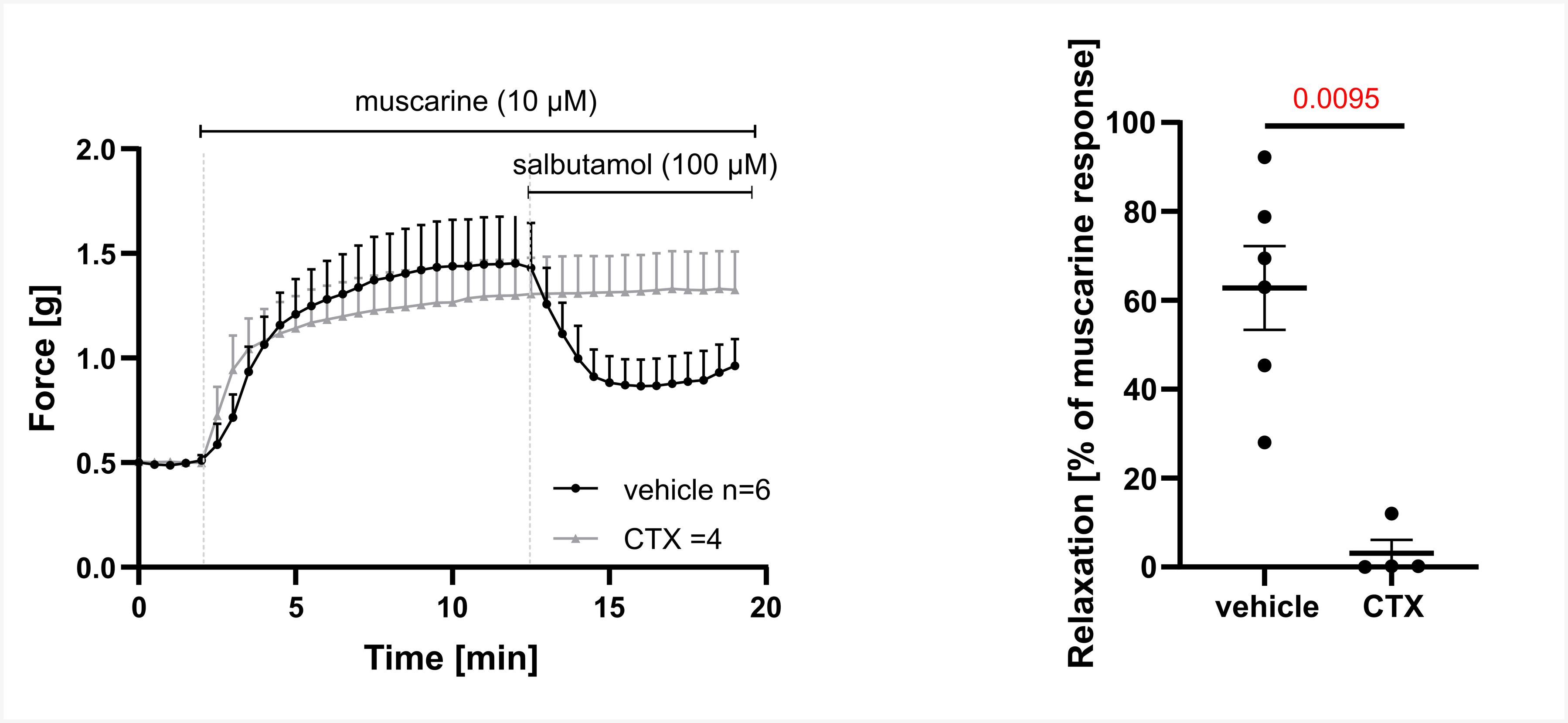

Supplement: S4 Fig — Force recordings from tracheal segments in an organ bath show that muscarine-induced constriction (10 µM) is set as 100%. Pretreatment with cholera toxin (CTX, 2 ng/ml) significantly attenuated salbutamol-induced relaxation of the tracheal segments. The scatterplot depicts β-NAD-induced relaxation as a percentage of the muscarine response in the absence (vehicle) and presence of CTX. N indicates the number of animals. Data are presented as means ± SEM, and statistical analysis was performed using the Mann-Whitney test. (TIF) [file pone.0334491.s004.tif]

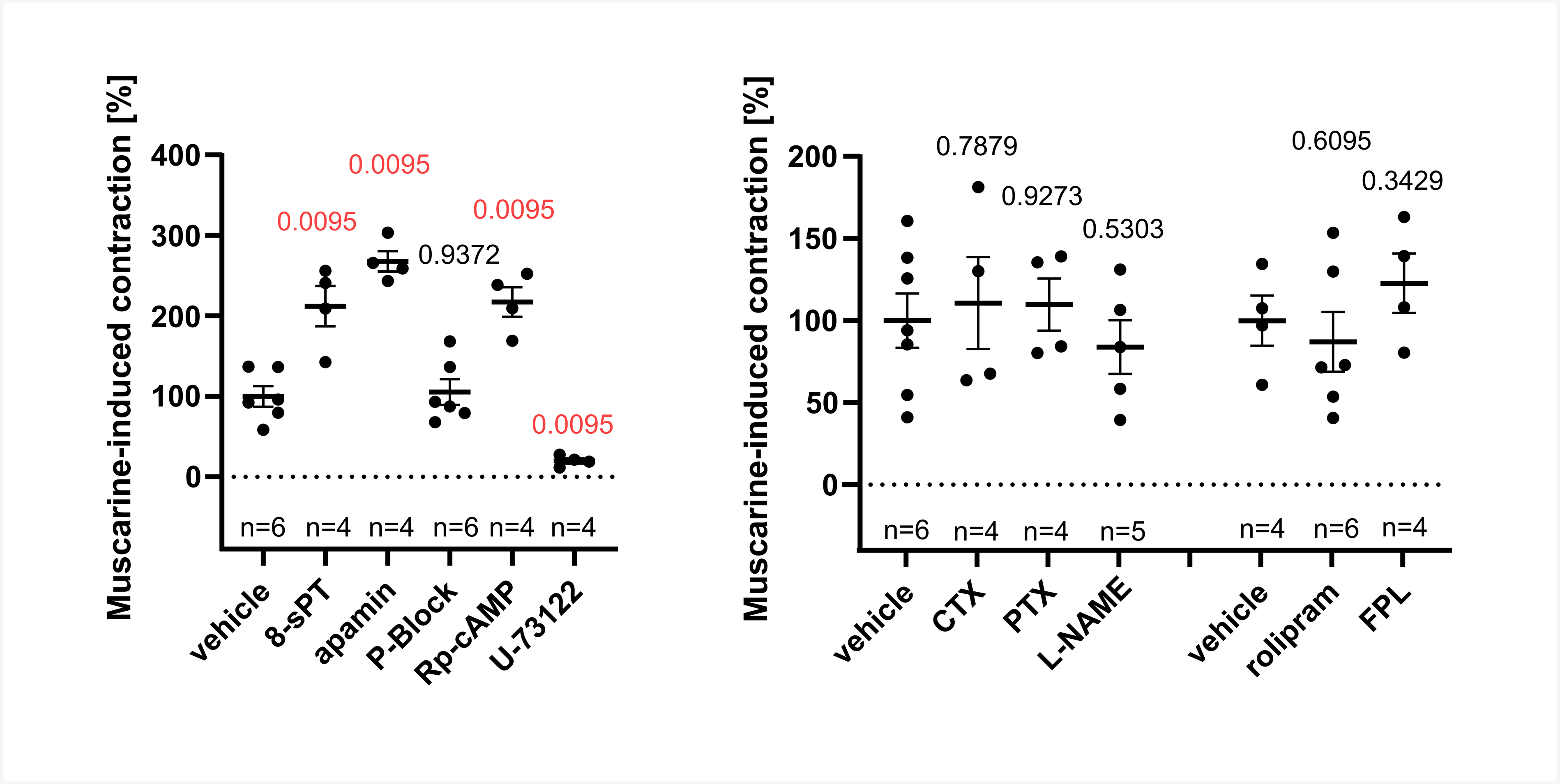

Supplement: S5 Fig — Muscarine-induced contraction data are normalized to the respective vehicle control for each experimental setup, with the vehicle response defined as 100%. The scatterplot displays the percentage of muscarine-induced contraction, with p-values shown above the data points (compared to the respective vehicle control, Kruskal-Walli’s test followed by Dunn’s multiple comparisons test). Whiskers represent the mean ± SEM. N indicates the number of animals. Substances tested include vehicle controls (0.03% DMSO, PBS, 0.01% DMSO, or 0.02% DMSO), purinergic receptor inhibitors (8-sPT, 10 µM; apamin, 10 µM; and P-Block comprising suramin, 100 µM, and PPADS, 30 µM), Rp-cAMP (100 µM), U-73122 (10 µM), cholera toxin (CTX, 2 ng/ml), pertussis toxin (PTX, 5 ng/ml), L-NAME (100 µM), rolipram (100 µM), and FPL64176 (10 µM). Among the tested substances, 8-sPT, apamin and Rp-cAMP significantly increased muscarine-induced contraction, while U-73122 inhibited muscarine responses. (TIF) [file pone.0334491.s005.tif]

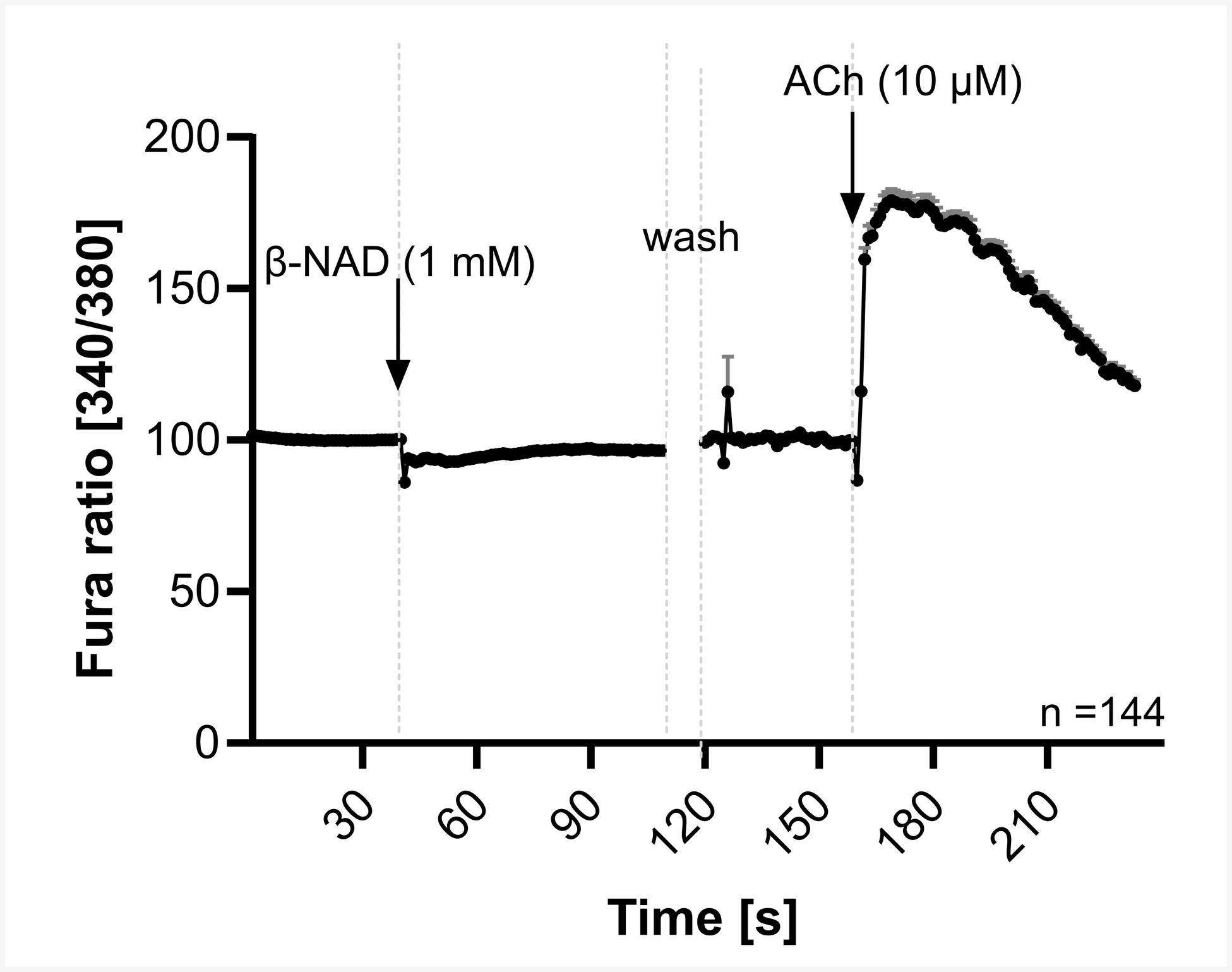

Supplement: S6 Fig — The Fura-2 340/380 fluorescence ratio of M3 muscarinic acetylcholine receptor-expressing M3WT4 cells is shown. The addition of β-NAD did not increase intracellular calcium levels, as indicated by the unchanged fluorescence ratio. Acetylcholine, used as a positive control at the end of the experiment, induced a robust increase, confirming the functionality of the receptor and the responsiveness of the assay. N indicates the number of cells. (TIF) [file pone.0334491.s006.tif]

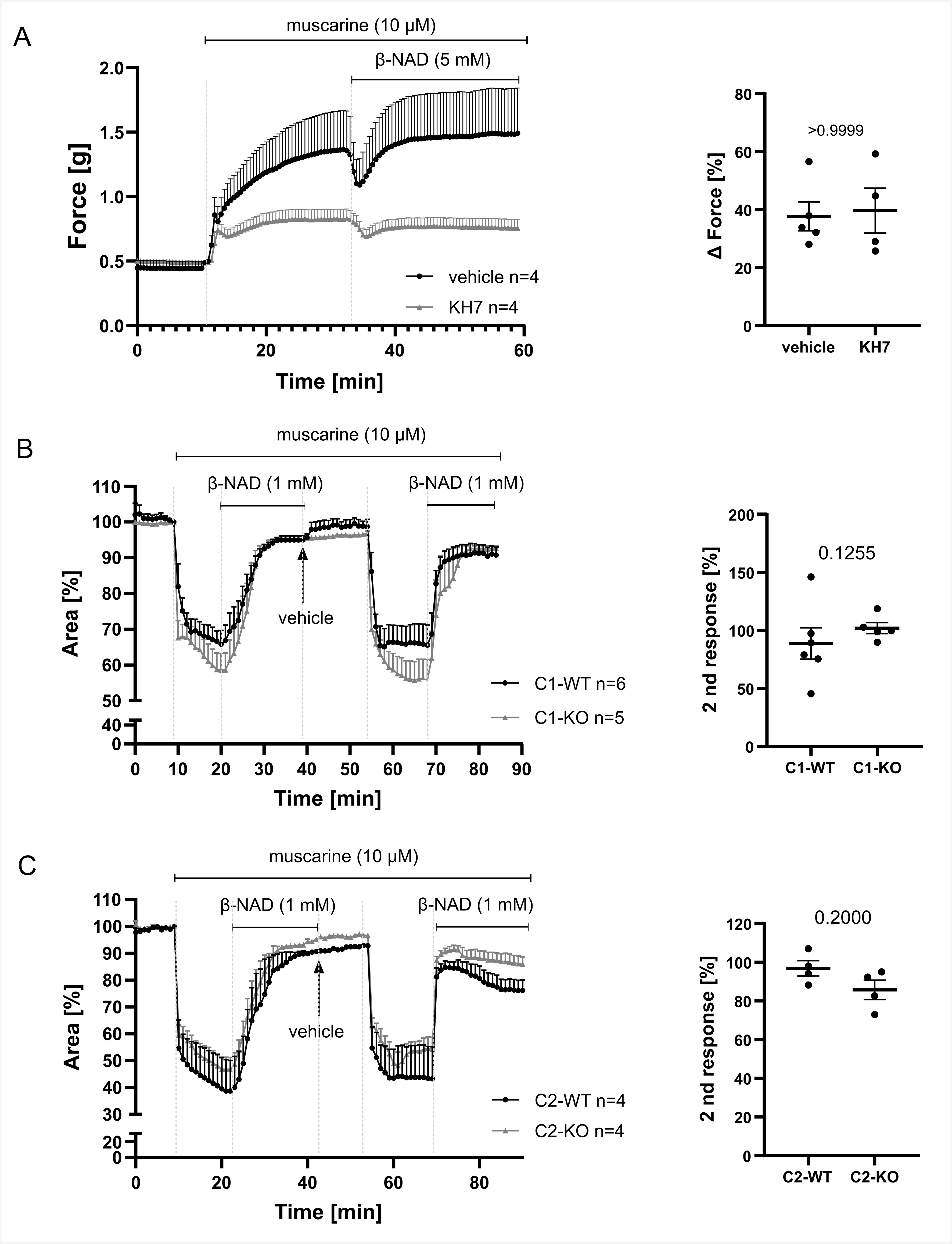

Supplement: S7 Fig — (A) Force recordings from tracheal segments in an organ bath preincubated with KH7 (30 µM; gray line), a SAC inhibitor dissolved in DMSO, or vehicle control (DMSO; final concentration: 0.003%; black line). β-NAD induced relaxation of muscarine-precontracted trachea in both KH7-treated and vehicle-treated tissues, with no significant difference observed between the two groups. (B, C) Videomorphometric recordings of the bronchial luminal area in PCLS, showing vehicle controls corresponding to Fig 6G and 6H. Two cycles of muscarine-induced contraction and β-NAD-induced relaxation were performed with vehicle (DMSO, 0.03%) applied between cycles. No significant differences in β-NAD-induced relaxation were observed between SAC C1 (B) or SAC C2 (C) knockout (KO) mice and their respective wild-type (WT) controls, nor were differences noted due to vehicle application. Scatterplot showing the maximum peak responses of the second stimulation (first response set as 100%) in the presence of the vehicle (DMSO treatment) within C1 and C2 knockout groups and their corresponding wild-type controls (B, C). Data are shown as means ± SEM. Statistical analysis was performed using the Mann-Whitney test. (TIF) [file pone.0334491.s007.tif]

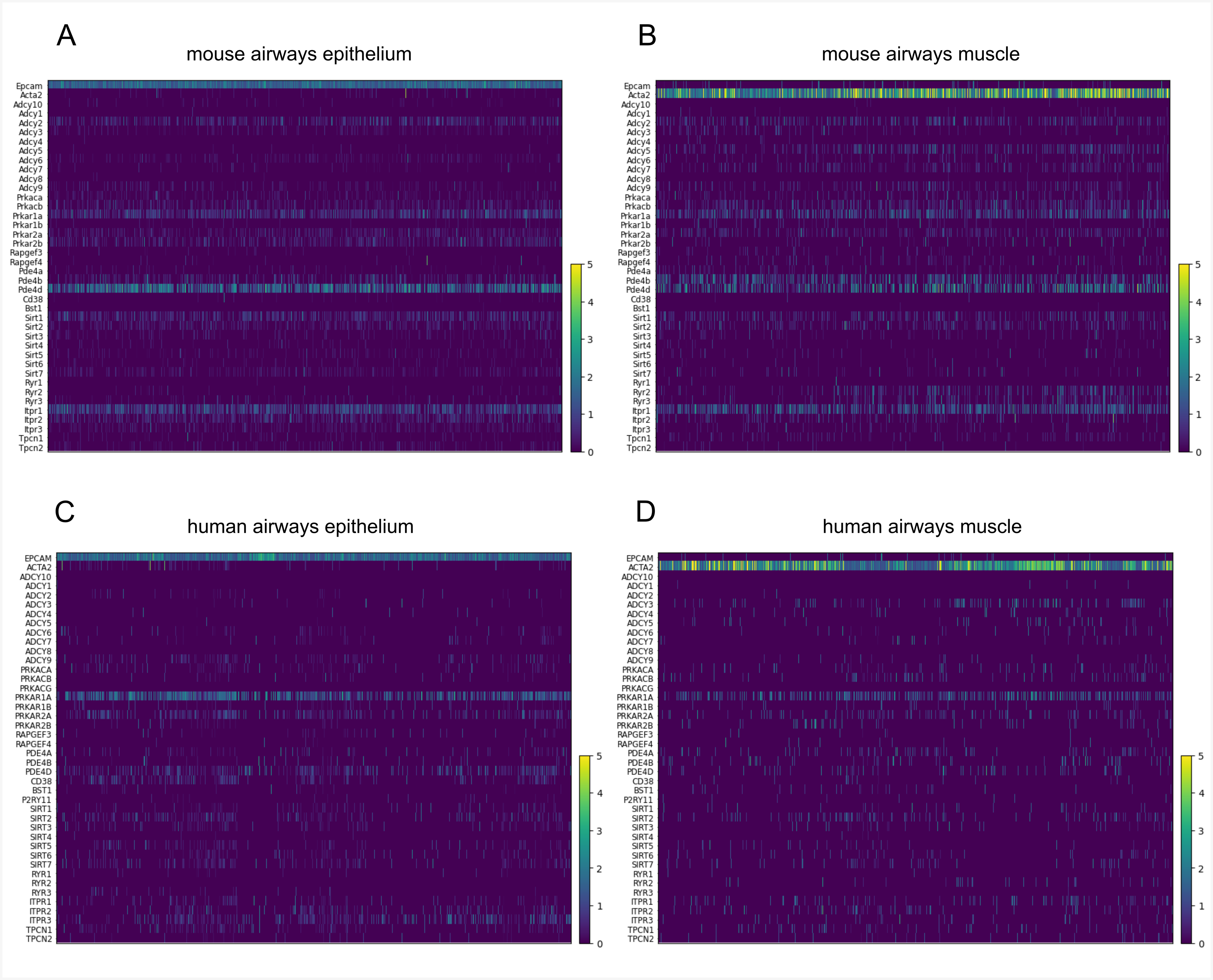

Supplement: S8 Fig — Single-cell RNA sequencing analysis of airway tissues from mice and human (datasets: GSE136831, GSE134174, GSE244215). (A) Mouse epithelial cells (EPCAM⁺). (B) Mouse airway smooth muscle cells (ACTA2⁺). (C) Human epithelial cells (EPCAM⁺). (D) Human airway smooth muscle cells (ACTA2⁺). Data are shown as UMI counts per cell. ADCY2, ADCY3, and ADCY9 are enriched in both epithelial and smooth muscle compartments. ADCY10 shows low expression in smooth muscle cells. PDE4B, PDE4D, RYR2, ITPR1, and PRKAR1A are expressed in both cell types, whereas ITPR3 is largely confined to epithelial cells. Gene symbols are presented in human nomenclature; corresponding mouse orthologs follow standard capitalization rules. (TIF) [file pone.0334491.s008.tif]

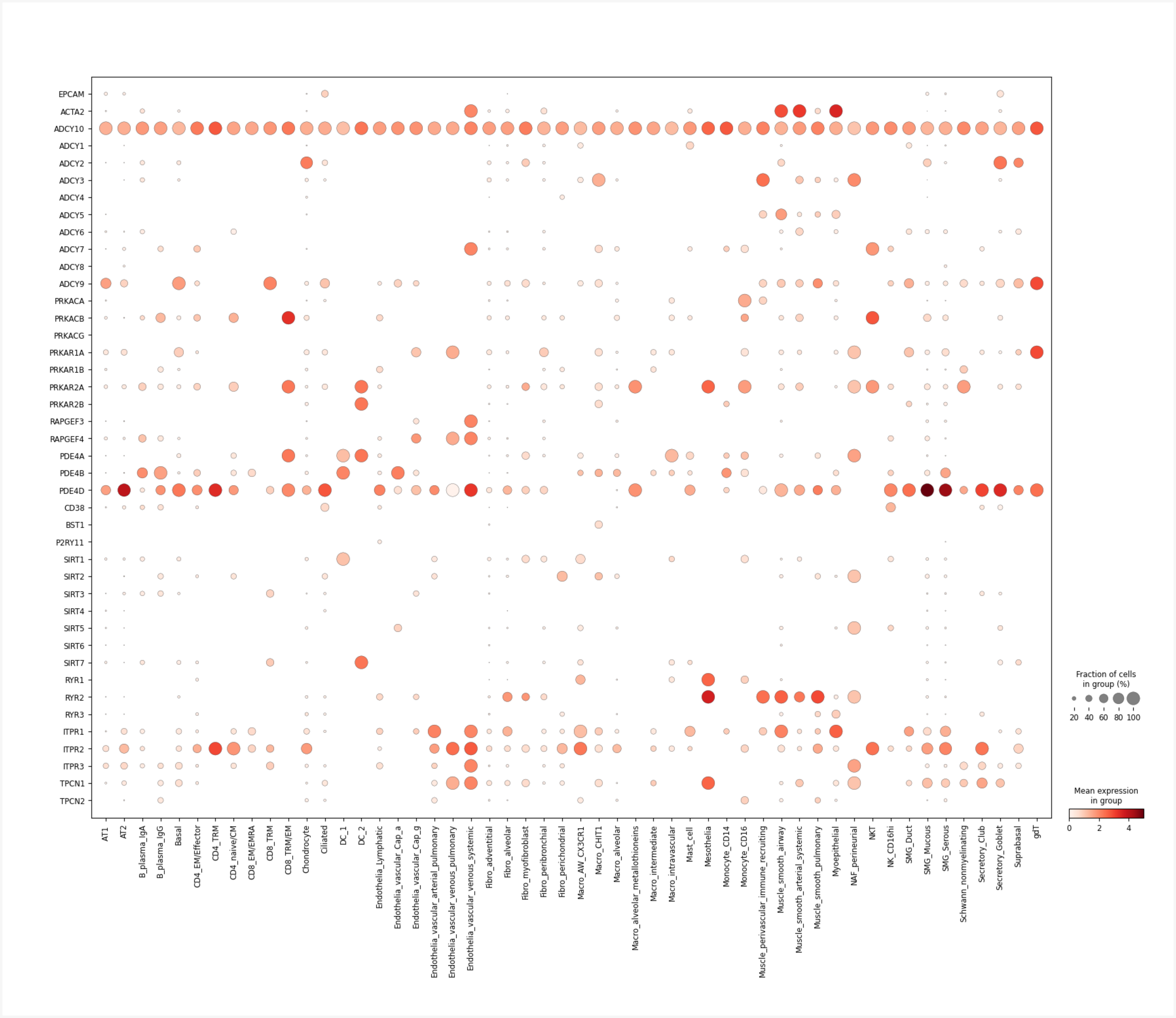

Supplement: S9 Fig — Single-cell and single-nucleus RNA-sequencing data from all major human airway cell types (datasets: GSE136831, GSE134174, GSE244215). Data are shown as UMI counts per cell. ADCY10 is broadly expressed across the dataset, while ADCY2 shows high expression in secretory goblet and chondrocyte cells. PDE4D, a phosphodiesterase relevant to β-NAD signaling, is widely expressed in both epithelial and mesenchymal compartments, supporting a role for phosphodiesterase regulation in airway smooth muscle relaxation. (TIF) [file pone.0334491.s009.tif]
