# Supplementary material for: β-Nicotinamide adenine dinucleotide (β-NAD) acts as a bronchodilator
Source: PLoS One. 2025 Oct 14;20(10):e0334491. doi: 10.1371/journal.pone.0334491 (PMC12520353; doi:10.1371/journal.pone.0334491)
Supplement: S1 Table — (DOCX) [file pone.0334491.s010.docx]

| **REAGENT or RESOURCE SOURCE IDENTIFIER** | | |
| --- | --- | --- |
| Minimal essential medium (MEM) | Thermo Fisher | Cat#51200038 |
| HEPES | Sigma Aldrich | Cat#H3375 |
| Sulfobromophthalein disodium salt hydrate | Sigma Aldrich | Cat#S0252 |
| Penicillin-Streptomycin | Sigma Aldrich | Cat#P4333 |
| Papain | Sigma Aldrich | Cat#P4762 |
| Ethylenediaminetetraacetic acid (EDTA•4Na) | Sigma Aldrich | Cat#E9884 |
| Poly-L-lysine | Sigma Aldrich | Cat#P-5899 |
| Laminin | Invitrogen | Cat#23017-015 |
| Fura-2-AM | Thermo Fisher | Cat#F1221 |
| RPMI 1640 Medium | Thermo Fisher | Cat#11875093 |
| Acetylcholine | Sigma Aldrich | Cat#A7000 |
| Locke's Solution | Elabscience | Cat#PB1 |
| Ham's F-12K (Kaighn's) Medium | Thermo Fisher | Cat#21127022 |
| Fetal Bovine Serum | Thermo Fisher | Cat#26140079 |
| Calcium Orange™, AM, cell permeant | Thermo Fisher | Cat#C3015 |
| β-Nicotinamidadenin-Dinucleotid Natriumsalz (β-NAD) | Sigma Aldrich | Cat#N0632 |
| Bovine serum albumin | Sigma Aldrich | Cat# 05470 |
| DTT (Diethiothreitol) | Thermo Fisher | Cat# R0861 |
| L-cysteine | Sigma Aldrich | Cat#14495 |
| leupeptin | Sigma Aldrich | Cat#62070 |
| Dispase® II | Sigma Aldrich | Cat# D4693 |
| fibronectin | Sigma Aldrich | Cat# F0895 |
| Smooth Muscle Cell Growth Medium 2 | PromoCell | Cat#C-22062 |
| Trypsin-EDTA | Thermo Fisher | Cat#25200056 |
| Sulfobromophthalein | Thermo Fisher | Cat#043878.03 |
| Anti-Actin, α-Smooth Muscle - FITC antibody, Mouse monoclonal | Sigma Aldrich | Cat# F3777 |
| DiBAC4(3) (Bis-(1,3-Dibutylbarbituric Acid)Trimethine Oxonol) | Invitrogen™ | Cat# B438 |
| Low melting point agarose | Sigma Aldrich | Cat# A9414 |
