# Supplementary material for: β-Nicotinamide adenine dinucleotide (β-NAD) acts as a bronchodilator
Source: PLoS One. 2025 Oct 14;20(10):e0334491. doi: 10.1371/journal.pone.0334491 (PMC12520353; doi:10.1371/journal.pone.0334491)
Supplement: S2 Table — (DOCX) [file pone.0334491.s011.docx]

| **DRUG** | **TARGET** | **FUNCTION** | **SOURCE** | **IDENTIFIER** |
| --- | --- | --- | --- | --- |
| Pyridoxalphosphate-6-azophenyl-2′,4′-disulfonic acid tetrasodium salt (PPADS) | purinergic receptors | general antagonist | Santa Cruz | Cat#sc-202770A |
| 8-(p-Sulfophenyl)theophylline hydrate (8-sPT) | adenosine receptor | antagonist | Sigma Aldrich | Cat#A013 |
| Adenosintriphosphate (ATP) | purinergic receptors | agonist | Sigma Aldrich | Cat#A9062 |
| Apamin | calcium-dependent kalium channels (SK-channels) | inhibitor | Santa Cruz | Cat# sc-200994 |
| Choleratoxin of *Vibrio cholerae* (CTX) | Gs-protein | activator | Sigma Aldrich | Cat#C8052 |
| Ethylenediaminetetraacetic acid (EDTA) | calcium | chelator | Santa Cruz | Cat#sc-204735A |
| FPL 64176 | L-type Ca²⁺ channel (nondihydro-pyridine Ca^2+^ channel) | activator | Sigma Aldrich | Cat#F131 |
| L-NG-Nitroarginine methylester (L-NAME) | NO-synthase | inhibitor | Sigma Aldrich | Cat#483125-M |
| MRS 2179 | P2Y1-receptors | antagonist | Sigma Aldrich | Cat#M3808 |
| Muscarine chloride | muscarinic receptor | agonist | Sigma Aldrich | Cat#M104 |
| Pertussistoxin of *Bordetella pertussis* (PTX) | Gi-protein | inhibitor | Merck | Cat#516560 |
| Rolipram | phosphodiesterase-4 (PDE-4) | inhibitor | Santa Cruz | Cat#sc-3563 |
| Rp-cAMP (Rp-adenosine 3′,5′-cyclic monophosphorothioate) | protein kinase A (PKA) | inhibitor | Santa Cruz | Cat#sc-24010 |
| Salbutamol | β2-adrenoceptor | agonist | Sigma Aldrich | Cat#S8260 |
| Suramin sodium | purinergic receptors | general antagonist | Santa Cruz | Cat#sc-507209F |
| U-73122 | phospholipase C (PLC) | inhibitor | Santa Cruz | Cat#sc-3574A |
