# Supplementary material for: β-Nicotinamide adenine dinucleotide (β-NAD) acts as a bronchodilator
Source: PLoS One. 2025 Oct 14;20(10):e0334491. doi: 10.1371/journal.pone.0334491 (PMC12520353; doi:10.1371/journal.pone.0334491)
Supplement: S3 Table — Expression of key cAMP signaling pathway genes in human and mouse airway epithelial (EPCAM⁺), smooth muscle (ACTA2⁺), and ADCY10 ⁺ cells was analyzed using single-cell RNA-seq datasets GSE136831, GSE134174, and GSE244215. Values represent the total number of cells expressing genes of interest with a UMI score > 1.0. The data highlight differential expression patterns of adenylyl cyclase isoforms (ADCY2/3/9 vs. ADCY10), phosphodiesterases (PDE4B/D), and cAMP effectors (PRKAR1A, RYR2, ITPR1) across species and cell types. (DOCX) [file pone.0334491.s012.docx]

| **Genes list** | **Human** | | | ***Genes list*** | **Old mouse** | | | **Young mouse** | | |
| --- | --- | --- | --- | --- | --- | --- | --- | --- | --- | --- |
|  | EPCAM^+^ | ACTA2^+^ | VIM^+^ |  | *Epcam^+^* | *Acta2^+^* | *Adcy10* | *Epcam^+^* | *Acta2^+^* | *Adcy10^+^* |
| EPCAM | 11059 | 284 | 2024 | *Epcam* | 5953 | 51 | 8 | 7170 | 65 | 12 |
| ACTA2 | 284 | 12013 | 8453 | *Acta2* | 51 | 1038 | 0 | 65 | 936 | 3 |
| VIM | 2024 | 8453 | 100922 | *Vim* | 457 | 561 | 27 | 558 | 701 | 59 |
| ADCY10 | 31 | 52 | 97 | *Adcy10* | 8 | 0 | 50 | 12 | 3 | 85 |
| ADCY1 | 24 | 132 | 241 | *Adcy1* | 8 | 1 | 0 | 10 | 7 | 0 |
| ADCY2 | 431 | 403 | 936 | *Adcy2* | 645 | 108 | 6 | 1462 | 107 | 19 |
| ADCY3 | 147 | 1352 | 3890 | *Adcy3* | 16 | 16 | 3 | 37 | 31 | 6 |
| ADCY4 | 51 | 255 | 5161 | *Adcy4* | 2 | 5 | 1 | 3 | 13 | 2 |
| ADCY5 | 44 | 900 | 543 | *Adcy5* | 4 | 114 | 1 | 14 | 122 | 2 |
| ADCY6 | 482 | 423 | 986 | *Adcy6* | 24 | 18 | 1 | 40 | 42 | 6 |
| ADCY7 | 301 | 414 | 5484 | *Adcy7* | 6 | 38 | 2 | 27 | 85 | 5 |
| ADCY8 | 97 | 30 | 44 | *Adcy8* | 1 | 0 | 0 | 1 | 0 | 0 |
| ADCY9 | 1752 | 1304 | 2902 | *Adcy9* | 37 | 31 | 6 | 97 | 68 | 9 |
| PRKACA | 277 | 556 | 4303 | *Prkaca* | 35 | 29 | 0 | 63 | 31 | 6 |
| PRKACB | 510 | 1228 | 7179 | *Prkacb* | 144 | 79 | 1 | 346 | 135 | 12 |
| PRKAR1A | 5062 | 3355 | 27075 | *Prkar1a* | 832 | 294 | 11 | 1790 | 418 | 37 |
| PRKAR1B | 295 | 451 | 4183 | *Prkar1b* | 7 | 9 | 1 | 14 | 16 | 3 |
| PRKAR2A | 1526 | 1590 | 8796 | *Prkar2a* | 97 | 59 | 3 | 237 | 93 | 7 |
| PRKAR2B | 196 | 541 | 1462 | *Prkar2b* | 345 | 22 | 1 | 539 | 25 | 4 |
| RAPGEF3 | 256 | 227 | 2610 | *Rapgef3* | 7 | 5 | 1 | 9 | 21 | 3 |
| RAPGEF4 | 335 | 295 | 2900 | *Rapgef4* | 28 | 24 | 6 | 44 | 36 | 8 |
| PDE4A | 381 | 695 | 6776 | *Pde4a* | 9 | 8 | 0 | 16 | 17 | 2 |
| PDE4B | 595 | 999 | 12718 | *Pde4b* | 343 | 267 | 7 | 661 | 374 | 33 |
| PDE4D | 3546 | 2413 | 10928 | *Pde4d* | 3789 | 399 | 20 | 4943 | 444 | 48 |
| CD38 | 902 | 234 | 2452 | *Cd38* | 5 | 2 | 0 | 6 | 5 | 0 |
| BST1 | 33 | 239 | 2857 | *Bst1* | 1 | 0 | 0 | 0 | 1 | 1 |
| P2RY11 | 75 | 101 | 1007 | *Ptprc* | 62 | 18 | 1 | 54 | 16 | 6 |
| SIRT1 | 396 | 520 | 3398 | *Sirt1* | 492 | 81 | 10 | 1084 | 143 | 20 |
| SIRT2 | 631 | 939 | 8324 | *Sirt2* | 91 | 55 | 5 | 193 | 95 | 14 |
| SIRT3 | 361 | 298 | 1778 | *Sirt3* | 40 | 14 | 1 | 72 | 18 | 2 |
| SIRT4 | 68 | 65 | 230 | *Sirt4* | 4 | 2 | 0 | 12 | 6 | 0 |
| SIRT5 | 356 | 345 | 1719 | *Sirt5* | 14 | 6 | 0 | 30 | 9 | 1 |
| SIRT6 | 226 | 335 | 2983 | *Sirt6* | 2 | 2 | 0 | 7 | 2 | 1 |
| SIRT7 | 452 | 520 | 5396 | *Sirt7* | 38 | 14 | 2 | 67 | 14 | 0 |
| RYR1 | 106 | 93 | 901 | *Ryr1* | 2 | 2 | 0 | 2 | 1 | 0 |
| RYR2 | 204 | 1631 | 1715 | *Ryr2* | 29 | 101 | 0 | 54 | 78 | 1 |
| RYR3 | 211 | 504 | 596 | *Ryr3* | 45 | 61 | 1 | 111 | 50 | 2 |
| ITPR1 | 1071 | 2522 | 7819 | *Itpr1* | 1823 | 402 | 29 | 3115 | 480 | 61 |
| ITPR2 | 1913 | 1829 | 10909 | *Itpr2* | 301 | 51 | 10 | 483 | 73 | 16 |
| ITPR3 | 1728 | 524 | 3491 | *Itpr3* | 42 | 14 | 4 | 88 | 17 | 6 |
| TPCN1 | 1069 | 1119 | 3852 | *Tpcn1* | 15 | 4 | 1 | 22 | 46 | 4 |
| TPCN2 | 164 | 415 | 2156 | *Tpcn2* | 48 | 4 | 0 | 183 | 9 | 2 |
